# *Tracembler *– software for *in-silico *chromosome walking in unassembled genomes

**DOI:** 10.1186/1471-2105-8-151

**Published:** 2007-05-09

**Authors:** Qunfeng Dong, Matthew D Wilkerson, Volker Brendel

**Affiliations:** 1 Department of Genetics, Development & Cell Biology, Iowa State University, Ames, Iowa 50011, USA; 2 Department of Statistics, Iowa State University, Ames, Iowa 50011, USA; 3 Center for Genomics and Bioinformatics, Indiana University, Bloomington, Indiana, USA

## Abstract

**Background:**

Whole genome shotgun sequencing produces increasingly higher coverage of a genome with random sequence reads. Progressive whole genome assembly and eventual finishing sequencing is a process that typically takes several years for large eukaryotic genomes. In the interim, all sequence reads of public sequencing projects are made available in repositories such as the NCBI Trace Archive. For a particular locus, sequencing coverage may be high enough early on to produce a reliable local genome assembly. We have developed software, *Tracembler*, that facilitates *in silico *chromosome walking by recursively assembling reads of a selected species from the NCBI Trace Archive starting with reads that significantly match sequence seeds supplied by the user.

**Results:**

*Tracembler *takes one or multiple DNA or protein sequence(s) as input to the NCBI Trace Archive BLAST engine to identify matching sequence reads from a species of interest. The BLAST searches are carried out recursively such that BLAST matching sequences identified in previous rounds of searches are used as new queries in subsequent rounds of BLAST searches. The recursive BLAST search stops when either no more new matching sequences are found, a given maximal number of queries is exhausted, or a specified maximum number of rounds of recursion is reached. All the BLAST matching sequences are then assembled into contigs based on significant sequence overlaps using the CAP3 program. We demonstrate the validity of the concept and software implementation with an example of successfully recovering a full-length *Chrm2 *gene as well as its upstream and downstream genomic regions from *Rattus norvegicus *reads. In a second example, a query with two adjacent *Medicago truncatula *genes as seeds resulted in a contig that likely identifies the microsyntenic homologous soybean locus.

**Conclusion:**

*Tracembler *streamlines the process of recursive database searches, sequence assembly, and gene identification in resulting contigs in attempts to identify homologous loci of genes of interest in species with emerging whole genome shotgun reads. A web server hosting *Tracembler *is provided at , and the software is also freely available from the authors for local installations.

## Background

Comparative genomics is based on the identification and alignment of homologous genes across multiple species and has become a standard, powerful approach in molecular biology for many purposes, including characterization of structurally and functionally important motifs in gene families. Typically, this approach starts with a set of query sequences as input to sequence similarity-based database search programs such as BLAST [1] to identify significantly similar matches in the sequence databases of species of interest. If the species of interest are fully sequenced and evolutionarily close enough to the query species, then this approach will yield the homologous genes in their genomic context. However, the comparative genomics approach is currently limited by the sparse sampling of eukaryotic species from the tree of life that have been sequenced as model organisms. For example, so far only three species (*Arabidopsis thaliana*, *Oryza sativa*, and *Populus trichocarpa*) from the entire plant kingdom have been completely sequenced. At the same time, a large number of on-going sequencing projects (see, e.g., [2,3]) are generating large numbers of short (yet unassembled) genomic sequences through strategies such as whole-genome shotgun or BAC-by-BAC minimum tiling path sequencing. These sequence reads are continuously made available through the NCBI Trace Archive [4]. In the summer of 2006 the archive topped one billion reads [5].

Because the deposited sequenced reads are short (400–800 bp), a simple query of the repository with a DNA or protein seed (e.g., NCBI's Trace Archive discontiguous Mega BLAST Server [6] or Ensembl's Trace Server [7]) will typically only tag this gene as present in the target genome. Depending on the genome sequence coverage, the query may result in redundant and overlapping tags. Analysis of the resulting set of reads without the help of an assembly program could become very tedious, particularly if one wishes to obtain the genome context of the tagged gene further upstream or downstream, which would require additional rounds of repository searches.

To facilitate the task of homolog identification in the trace archive repository of an ongoing genome sequencing project, we have developed software that seamlessly integrates recursive database searches and contig assembly and interpretation. Depending on the depth of the current sequencing effort, the final results returned by *Tracembler *will ideally be full-length genomic sequences that are homologs of the user-supplied query sequences.

## Implementation

**Figure 1:**
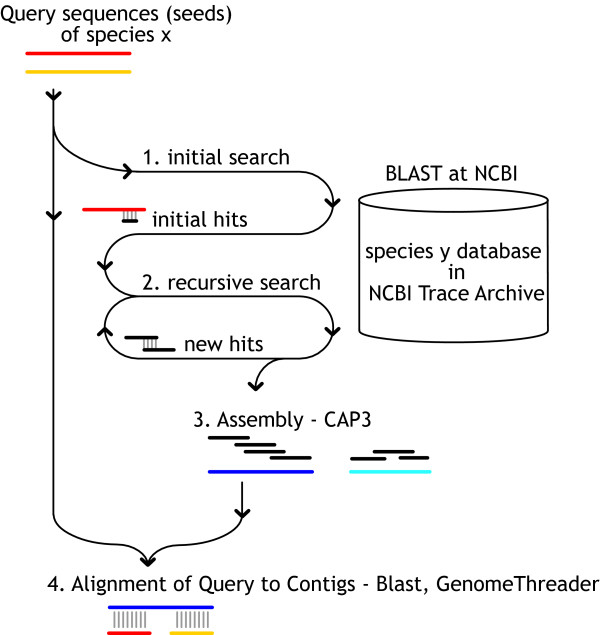
**Schematic overview of the *Tracembler *algorithm**. Tracembler accepts as input one or more user-supplied query sequences and parameter specifications. The query sequence(s) and associated parameters are submitted using the QBLAST URL API to NCBI [25] (1). Tracembler analyzes these results, and if there are new sequences matching at a significance level below the user supplied E-value parameter, these sequences are used as queries in a new BLAST search (2). One round consists of BLAST searches of all acceptable matching sequences from the previous round. This process is repeated in a recursive manner until either all matching sequences are exhausted, a user-defined maximum round of recursion is reached, or a user defined maximum total number of BLAST queries is reached. For the final set of sequences, quality score and mate-pair distance constraint information is retrieved from NCBI. These sequences are assembled using CAP3 (3). Finally, novel contigs are compared to the query sequences using BLAST for local alignment and GenomeThreader for spliced-alignment (4).

The *Tracembler *algorithm is illustrated in Fig. 1. As input, the program takes a single or multiple user-supplied query sequences (either nucleotide or protein), an E-value cutoff, and a user selected Trace Archive database, which contains sequence reads from a particular species deposited at the NCBI Trace Archive. An initial search is then initiated via the remote BLAST service provided by NCBI [6,8]. Because the searches are always performed directly at NCBI (conducted transparently to the user), users are assured to search against the most up-to-date sequence read repository. If the initial BLAST search returns significant matching sequences (based on the user-specified E-value cutoff), these matching sequences are considered as queries for further database searches, which can potentially extend the initial matching region in both 5' and 3' direction (*in-silico *chromosome walking). This process is automatically iterated until either no more new matching sequences are found, a given maximal number of queries is exhausted, or a specified maximum number of rounds of recursion is reached. Only newly identified non-redundant matching sequences from the previous round are used as queries for the next round BLAST search. The ceiling on the number of rounds of recursion is imposed to prevent assembly of more than the local regions surrounding the genes of interest, thus protecting the intellectual properties of the whole-genome sequencing project by preventing large-scale assembly (e.g., [9]). Additionally, *Tracembler *has the following rules for polite dynamic NCBI data requests: mandatory pauses between data requests and BLAST job submissions, a maximum number of requests for an individual BLAST result, fixed limits on the BLAST parameter values.

All the obtained BLAST matching sequences are considered as potential genomic constituents of homologous regions of the original user-supplied query sequence and are assembled with the CAP3 program [10]. In addition to the actual sequences, quality scores and mate-pair distance constraints are also critical for high-quality assembly. Therefore, the quality score and mate-pair distance constraint information for each read are dynamically retrieved from the Trace Archive and used in the assembly to evaluate the significance of sequence overlaps. Multiple contigs may result from coverage gaps in one locus or represent duplicated loci. The CAP3-generated contigs are compared with the original user-supplied query sequences using BLAST [1] and *GenomeThreader *[11] to assess and display the extent of similarity and coverage. After completion of the analysis, an email is sent to the user indicating URLs to view all the results, including the assembled contig sequences, the multiple-sequence alignment underlying the assembly, as well as the pair-wise alignments between the original query and the contigs. In addition, the intermediate files (matching sequences, quality scores, and mate-pair distance constraints), the recursive BLAST output, the CAP3 output files, and a log file are included in the result. These additional files permit interested users to download and locally analyze their data further, such as using a different assembly program.

## Results and discussion

### Validation

To validate *Tracembler*, we first tested the software by trying to re-assemble a published genome region from trace reads matching an annotated gene. Our test case used the rat (*Rattus norvegicus*) *Chrm2 *gene sequence (cholinergic receptor, muscarinic 2; 2,072 bp; [12]) as query against the entire rat whole-genome shotgun sequence reads. The gene was picked randomly. The rat genome has already been fully sequenced and assembled [13], and thus *Tracembler *was expected to assemble a contig that matches the published genome. As shown in "**Additional file **1: ***Tracembler *validation and applications**", *Tracembler *produced a single contig of length 5,068 bp. This contig covers the entire, perfectly matched *Chrm2 *gene. The entire contig matches very well to chromosome 4 (GenBank accession# NC_005103.2) from base positions 63,909,839 to 63,914,888 (99% identity over the entire match as reported by bl2seq [14]). The *Chrm2 *gene maps from base positions 63,911,288 to 63,913,359 [15], and thus the contig generated by *Tracembler *not only recovered the full-length annotated *Chrm2 *gene but also successfully "walked" 1,449 bp into the 5' upstream and 1,529 bp into the 3' down-stream regions.

### Application

Next, we discuss a *Tracembler *application that revealed microsynteny between *Medicago truncatula *and *Glycine max*, which are thought to have diverged through speciation around 50 MYA [16]. *M. truncatula *is an established model organism for the legumes, with a nearly complete sequencing and annotation effort [17]. Whole genome shot-gun sequencing of *G. max *(soybean) has only recently been initiated [18], with currently more than 1.3 million unassembled and unannotated sequence reads deposited in the NCBI Trace Archive. Soybean is the most valuable legume crop [19], and establishing its syntenic relationship with *M. truncatula *is critical for transferring knowledge from this model organism.

In *M. truncatula*, the "SWIM zinc finger" gene (AC146590g10v2) is annotated on a BAC clone (mth2-145p10) from position 50,413 to 49,886 [20]. 3' downstream of this gene, there is another "hypothetical" gene (AC146590g11v2) annotated from positions 52,448 to 50,777. According to the current *M. truncatula *genome annotation, the "SWIM zinc finger" gene and its neighboring "hypothetical" gene are only 364 bp apart. In order to investigate whether such close distance is likely a result of mis-annotation ("hypothetical" genes are often wrongly predicted by gene-finding software), we took the protein sequences encoded by these two *M. truncatula *genes as input and used *Tracembler *to search against soybean sequence reads at NCBI Trace Archive. Interestingly, one 4,172 bp soybean genomic contig obtained from the assembly does match well to both the "SWIM zinc finger" and the "hypothetical" protein from positions 1,459 to 1,950 and from positions 2,299 to 2,839 of this contig, respectively (see **Additional file **1: ***Tracembler *validation and applications**). Thus, there appear to be homologs of the Medicago genes on the soybean genome in similar proximity (349 bp apart) as on the Medicago genome. Our result provides compelling evidence that the two genes are highly conserved between *M. truncatula *and soybean. In particular, the high conservation of the "hypothetical" gene suggests that it is a true gene.

### Performance

The performance of *Tracembler *is mainly determined by three factors. The first is the sequencing depth of the target genome, which provides a boundary of the expected extent of read overlaps and therefore assembled contig length. The second factor is the abundance of gene duplications in the genome of interests. For plant genomes, in which many gene duplications have occurred through tandem or whole genome duplication events, multiple homologs of genes of interests may have been sampled by the deposited sequence reads and show up as close BLAST matching sequences in the initial stage of *Tracembler*. If the multiple gene copies are sufficiently diverged, the CAP3 program will split them into different contigs. The pairwise comparison between the original user-supplied queries and the final contigs in the final step of *Tracembler *may identify the likely ortholog of the query based on highest match score. Third, because Tracembler relies on the up-to-date NCBI Trace Archive BLAST search engine over the Web, the response time for users will be affected by network traffic as well as the current work-load at the search engine. Various parameter settings deal with the stringency of matching and extent of the search, which will also affect speed and quality of the results.

### Other programs

During the preparation of this manuscript, we became aware of a published software package, *GENOTRACE*, from Berezikov *et al*. [21] that is similar to our *Tracembler*. In addition to the choices of embedded external computer programs (e.g., BLAST vs. SSAHA [22] for database searching; CAP3 *vs*. Phrap [23] for assembly) that likely produce different final outputs, there are several subtle yet important differences between *Tracembler *and *GENOTRACE *that matter to the general biology user community. First, *GENOTRACE *requires maintaining a local copy of NCBI Trace Archive. Although this approach improves the search speed, the required amount of disk space is enormous (currently more than 1.2 TBytes in compressed format at NCBI), which is beyond a typical user's resources and is superfluous for the task of exploring just a few genes of interest. By contrast, *Tracembler *takes advantage of the dynamic API provided by NCBI and sends query sequences via the internet to directly search the Trace Archive at NCBI. This not only eases the installation and maintenance for the users, but also ensures that users are always searching the most up-to-date version of Trace Archive. Furthermore, because trace sequences can often accumulate in amounts of hundreds of thousands of sequences per organism per week [24], *GENOTRACE*'s requirement of a local copy of NCBI's Trace archive necessitates frequent downloading and processing of local files, which is an obstacle for widespread use. Second, only DNA sequence can be used as query for *GENOTRACE*, whereas *Tracembler *can take either DNA or protein sequences as input (the program automatically detects the type of sequences and performs appropriate BLAST-searches, MEGABLAST or TBLASTN, accordingly). Third, *GENOTRACE *is restricted to one query sequence at a time. As demonstrated by our application example above, there are instances where it is more convenient to allow multiple seeds spanning one region of interest in one genome to search another genome.

## Conclusion

Biologists are often left with an eager sense of anticipation when their species of interest are in the process of being sequenced but the sequencing reads have not yet been assembled. Our *Tracembler *server, although algorithmically simple, provides an elegant solution for biologists to recover genomic regions of interest from species with on-going sequencing project before the whole genome assemblies are published.

## Availability and requirements

The *Tracembler *program is freely accessible, using a web browser at . The software, written in Perl and designed for use on Linux machines, is also freely available for local installation by download from . Instructions on obtaining the required external free programs (in particular, CAP3 [10]) are provided with the software.

## Authors' contributions

VB designed and supervised the project. QD and MW implemented the computer program. QD analyzed the results and prepared the manuscript. All authors read and approved the final manuscript.

## Supplementary Material

Additional file 1 *Tracembler *validation and applications. Examples of *Tracembler *usage Click here for file

## References

[B1] Altschul SF, Madden TL, Schäffer AA, Zhang J, Zhang Z, Miller W, Lipman DJ (1997). Gapped BLAST and PSI-BLAST: a new generation of protein database search programs. Nucleic acids research.

[B2] NCBI Entrez Genome Project. http://www.ncbi.nlm.nih.gov/entrez/query.fcgi?db=genomeprj.

[B3] JGI Sequencing Plans and Progress. http://www.jgi.doe.gov/sequencing/seqplans.html.

[B4] NCBI Trace Archive. http://www.ncbi.nlm.nih.gov/Traces/trace.cgi.

[B5] National Center for Biotechnology Information Newsletter 15(1), Summer 2006. http://www.ncbi.nlm.nih.gov/Web/Newsltr/V15N1/trace.html.

[B6] NCBI Trace Archive discontiguous Mega BLAST Server. http://www.ncbi.nlm.nih.gov/blast/tracemb.shtml.

[B7] Ensembl Trace Server. http://trace.ensembl.org/cgi-bin/tracesearch.

[B8] McGinnis S, Madden TL (2004). BLAST: at the core of a powerful and diverse set of sequence analysis tools. Nucleic acids research.

[B9] NHGRI Rapid Data Release Policy. http://www.genome.gov/10506376.

[B10] Huang X, Madan A (1999). CAP3: A DNA sequence assembly program. Genome research.

[B11] Gremme G (2005). Engineering a software tool for gene structure prediction in higher organisms.. Information Software Technol.

[B12] Rattus norvegicus Chrm2, cholinergic receptor muscarinic 2 gene sequence. http://www.ncbi.nlm.nih.gov/entrez/viewer.fcgi?val=NC_005103.2&from=63911288&to=63913359&dopt=fasta.

[B13] Gibbs RA, Weinstock GM, Metzker ML, Muzny DM, Sodergren EJ, Scherer S, Scott G, Steffen D, Worley KC, Burch PE, Okwuonu G, Hines S, Lewis L, DeRamo C, Delgado O, Dugan-Rocha S, Miner G, Morgan M, Hawes A, Gill R, Holt RA, Adams MD, Amanatides PG, Baden-Tillson H, Barnstead M, Chin S, Evans CA, Ferriera S, Fosler C, Glodek A, Gu Z, Jennings D, Kraft CL, Nguyen T, Pfannkoch CM, Sitter C, Sutton GG, Venter JC, Woodage T, Smith D, Lee HM, Gustafson E, Cahill P, Kana A, Doucette-Stamm L, Weinstock K, Fechtel K, Weiss RB, Dunn DM, Green ED, Blakesley RW, Bouffard GG, De Jong PJ, Osoegawa K, Zhu B, Marra M, Schein J, Bosdet I, Fjell C, Jones S, Krzywinski M, Mathewson C, Siddiqui A, Wye N, McPherson J, Zhao S, Fraser CM, Shetty J, Shatsman S, Geer K, Chen Y, Abramzon S, Nierman WC, Havlak PH, Chen R, Durbin KJ, Egan A, Ren Y, Song XZ, Li B, Liu Y, Qin X, Cawley S, Worley KC, Cooney AJ, D'Souza LM, Martin K, Wu JQ, Gonzalez-Garay ML, Jackson AR, Kalafus KJ, McLeod MP, Milosavljevic A, Virk D, Volkov A, Wheeler DA, Zhang Z, Bailey JA, Eichler EE, Tuzun E, Birney E, Mongin E, Ureta-Vidal A, Woodwark C, Zdobnov E, Bork P, Suyama M, Torrents D, Alexandersson M, Trask BJ, Young JM, Huang H, Wang H, Xing H, Daniels S, Gietzen D, Schmidt J, Stevens K, Vitt U, Wingrove J, Camara F, Mar Alba M, Abril JF, Guigo R, Smit A, Dubchak I, Rubin EM, Couronne O, Poliakov A, Hubner N, Ganten D, Goesele C, Hummel O, Kreitler T, Lee YA, Monti J, Schulz H, Zimdahl H, Himmelbauer H, Lehrach H, Jacob HJ, Bromberg S, Gullings-Handley J, Jensen-Seaman MI, Kwitek AE, Lazar J, Pasko D, Tonellato PJ, Twigger S, Ponting CP, Duarte JM, Rice S, Goodstadt L, Beatson SA, Emes RD, Winter EE, Webber C, Brandt P, Nyakatura G, Adetobi M, Chiaromonte F, Elnitski L, Eswara P, Hardison RC, Hou M, Kolbe D, Makova K, Miller W, Nekrutenko A, Riemer C, Schwartz S, Taylor J, Yang S, Zhang Y, Lindpaintner K, Andrews TD, Caccamo M, Clamp M, Clarke L, Curwen V, Durbin R, Eyras E, Searle SM, Cooper GM, Batzoglou S, Brudno M, Sidow A, Stone EA, Venter JC, Payseur BA, Bourque G, Lopez-Otin C, Puente XS, Chakrabarti K, Chatterji S, Dewey C, Pachter L, Bray N, Yap VB, Caspi A, Tesler G, Pevzner PA, Haussler D, Roskin KM, Baertsch R, Clawson H, Furey TS, Hinrichs AS, Karolchik D, Kent WJ, Rosenbloom KR, Trumbower H, Weirauch M, Cooper DN, Stenson PD, Ma B, Brent M, Arumugam M, Shteynberg D, Copley RR, Taylor MS, Riethman H, Mudunuri U, Peterson J, Guyer M, Felsenfeld A, Old S, Mockrin S, Collins F, Celera (2004). Genome sequence of the Brown Norway rat yields insights into mammalian evolution. Nature.

[B14] NCBI bl2seq Web Server. http://www.ncbi.nlm.nih.gov/blast/bl2seq/wblast2.cgi.

[B15] NCBI Entrez Gene Chrm2 Region. http://www.ncbi.nlm.nih.gov/entrez/query.fcgi?db=gene&cmd=Retrieve&dopt=full_report&list_uids=81645.

[B16] Mudge J, Cannon SB, Kalo P, Oldroyd GE, Roe BA, Town CD, Young ND (2005). Highly syntenic regions in the genomes of soybean, Medicago truncatula, and Arabidopsis thaliana. BMC plant biology.

[B17] Young ND, Cannon SB, Sato S, Kim D, Cook DR, Town CD, Roe BA, Tabata S (2005). Sequencing the genespaces of Medicago truncatula and Lotus japonicus. Plant physiology.

[B18] Department of Energy Press Release. http://www.energy.gov/news/2979.htm.

[B19] Wilson RF, Stalker HT, Brummer C (2004). Legume Crop Genomics.

[B20] Medicago truncatula IMGAG Genome Annotation. http://www.tigr.org/tigr-scripts/medicago/IMGAG/tab_delimited_output?word=&locus=AC146590_10.2&accession=.

[B21] Berezikov E, Plasterk RH, Cuppen E (2002). GENOTRACE: cDNA-based local GENOme assembly from TRACE archives. Bioinformatics (Oxford, England).

[B22] Ning Z, Cox AJ, Mullikin JC (2001). SSAHA: a fast search method for large DNA databases. Genome research.

[B23] Phrap. http://www.phrap.org.

[B24] NCBI Trace Archive Statistics. http://www.ncbi.nlm.nih.gov/Traces/trace.cgi?cmd=show&f=graph_query&m=stat&s=graph.

[B25] NCBI QBlast's URL API. User's Guide. http://www.ncbi.nlm.nih.gov/BLAST/Doc/urlapi.html.

