# Supplementary material for: Tracembler – software for in-silico chromosome walking in unassembled genomes
Source: BMC Bioinformatics. 2007 May 9;8:151. doi: 10.1186/1471-2105-8-151 (PMC1876249; doi:10.1186/1471-2105-8-151)
Supplement: Additional file 1 — Tracembler validation and applications. Examples of Tracembler usage [file 1471-2105-8-151-S1.htm]

Examples for Tracembler


### *Tracembler* Examples - the additional datafiles for the submitted paper:

"***Tracembler* - software for *in-silico* chromosome walks in unassembled genomes**  
by Qunfeng Dong, Matthew D. Wilkerson, and Volker Brendel"  

The following examples were produced using the *Tracembler* Web Server (http://www.plantgdb.org/tool/tracembler/) on Jan 2, 2007.

**Example 1: Recovering rat chrm2 gene and its 5' and 3' upstream regions**

- *chrm2* gene sequence used as query
- Parameters being used
- The contig sequence assembled by Tracembler
- Pairwise alignment between the original *chrm2* sequence and the obtained contig
- Pairwise alignment between the obtained contig and entire rat Chromosome 4

**Example 2: Micro-synteny of two genes between Medicago truncatula and soybean**

- Two *Medicago truncatula* proteins used as query
- Parameters being used
- The produced contig that covers the soybean homologs of the above two Medicago proteins
- Pairwise alignment between the original Medicago query sequences and the above contig
- Spliced alignment between the above contig and the two query protein sequences

[Back to the top]

### Example 1. Recovering rat *chrm2* gene and its 5' and 3' upstream regions

[Back to the top]

**A. *chrm2* gene sequence used as query**

```
>ref|NC_005103.2|NC_005103:63911288-63913359 Rattus norvegicus cholinergic receptor, muscarinic 2 (Chrm2)
CCCTACAGGTTTAAATGTTTCTTTGGCCACTTGACTACTGAACACAAAATGAATAACTCAACAAACTCCT
CGAACAATGGCTTGGCTATTACCAGTCCTTACAAGACATTTGAAGTGGTATTTATTGTCCTTGTGGCTGG
ATCCCTCAGTCTGGTGACCATCATTGGGAACATTCTGGTCATGGTTTCCATTAAAGTCAACCGCCACCTT
CAGACTGTCAACAATTACTTCTTGTTCAGCCTGGCCTGTGCTGACCTCATCATAGGTGTTTTCTCCATGA
ACTTGTATACCCTCTACACTGTGATTGGTTACTGGCCTTTGGGACCTGTAGTATGTGACCTTTGGCTAGC
ATTGGACTATGTTGTCAGCAATGCCTCCGTTATGAATCTCCTCATCATCAGCTTTGATAGATACTTCTGT
GTCACGAAACCTCTGACCTACCCAGTTAAGCGGACCACAAAAATGGCAGGCATGATGATTGCAGCTGCGT
GGGTCCTTTCCTTCATCCTCTGGGCCCCAGCCATTCTCTTCTGGCAGTTCATCGTAGGGGTGAGGACTGT
GGAGGATGGGGAGTGCTATATTCAGTTCTTTTCCAATGCGGCCGTCACCTTCGGCACTGCCATTGCAGCT
TTCTATCTGCCTGTCATCATCATGACTGTGCTCTATTGGCATATATCCCGGGCAAGCAAGAGTAGAATAA
AGAAGGAAAAGAAGGAACCTGTGGCCAACCAAGACCCAGTATCTCCAAGTCTGGTGCAAGGAAGAATTGT
AAAGCCAAACAATAACAATATGCCTGGTGGTGATGGCGGCCTGGAACACAACAAGATCCAGAATGGCAAG
GCTCCACGGGACGGCGTGACTGAAAACTGTGTTCAGGGGGAGGAGAAAGAGAGCTCCAATGATTCGACGT
CAGTCAGTGCTGTGGCCTCCAATATGAGAGATGATGAGATAACCCAGGATGAAAACACAGTTTCCACTTC
GCTGGGCCACTCCAGAGATGACAACTCTAAGCAAACATGCATCAAAATTGTCACCAAGGCCCAAAAGGGT
GATGTGTGCACCCCAACGAGTACCACTGTAGAACTAGTTGGGTCGTCGGGTCAGAATGGGGATGAAAAGC
AGAACATTGTAGCCCGCAAAATCGTGAAGATGACCAAGCAGCCTGCCAAAAAGAAGCCTCCACCATCCCG
GGAAAAGAAAGTGACCAGGACAATCTTGGCTATCCTGTTGGCTTTCATCATAACGTGGGCGCCATACAAT
GTCATGGTGCTCATCAATACTTTCTGTGCACCCTGCATCCCCAATACAGTATGGACAATTGGCTACTGGC
TCTGTTACATCAATAGCACCATCAATCCGGCCTGCTATGCGCTTTGTAATGCCACCTTCAAAAAGACTTT
TAAGCACCTCCTCATGTGTCATTACAAGAACATAGGCGCTACACGGTGAAAAGACCATCAAAAGAAGAAA
TGTGGTCGAGTGTGTCTTGGGGAAGAACAGAGACAAGAAAGCTGTGTTTATAGTGACCTGCCATTTCACT
TTACAGTCTTACTGCAACATGAAAGTAAGGAGTTTTAGAGAGACACTATCATTGTGCCCATGCTCCATTT
TGGGAAAAATAAATTAATAAACCTTCACCTTATAAACCCTGTCAGTTTAGGAGCACCGAGAAAATGAAAG
AGGCATGCTGAAACTGCAGATCTAAGGAAAAATCTCTACTGTCTCCTGCTCTCTTGAAGAAGGGCGTCAG
AGTCTACAATTTCATGTCTCTGCACAAGAAGAATAACCTAGTCTATTTGTTGTTTCTTTCTGTTGTTCCC
CTGTGTGGCGGTGAGAAAGAAATGACACATTCCATGCTAACACAGAGACCTACATGGAAAGAAGCAGGCA
CTGTACAATGAGAGAGAAGAAGAAAGAAAATCAAATAGGATGCAGAGATGGTCTGCAGAGCAGCGAGCAT
ATCCTCGGCTGTGCTGTGCTCTGATCTGAAGGATTTCAACACAACAACTGCTTATTTCTTCTCTCTTCCC
CTTTCCCGTCATGGAAAGCAAGAAAGCAAAACAACAAAAGCC
```

[Back to the top]

**B. Parameters being used**

```
        Database: Rattus_norvegicus_WGS
        Initial E-value: 1e-100
        Round E-value: 1e-60
        Round Limit: 3
        Max Queries: 50
        Initial BLAST
                Percent Identity: none
                Filters: Low Complexity, Mask for lookup table only
        Recursive BLAST
                Word Size: 32
                Percent Identity: none
                Filters: Low Complexity, Rodents Repeats, Mask for lookup table only
        GenomeThreader Species: rat
```

[Back to the top]

**C. The contig sequence assembled by *Tracembler***

```
>Contig
AAGCTGGTGGAAGCCAGCTACTATCTCTCATAAAAAAGGAACAGCCTTCACATAATGTGT
GCATCCCCTTGGAAGATGGGATGTCCCTGCTTCGAATACTTGCGCTTGCTTCTAAATGGC
ATGTTTCCCATTGTCACACCAGTACTGCCACAGACCAGTAGGGCGTCTCTAAAGATTCTG
AGCTGCAATAGTCAATATAGTTCCCGTCCCATAGAGTTTTCAGTGTTCAATATGGATAAG
GATCCCAGGTGCATAAACTCTCCGTTTTGACTAGCAACGTCTGCACATAAGGCATCATCA
TGTTGCTCCTCGGGAATCTCTCATTAAGTGCAACCCTTCAATGGAAAAGGGAGGCAGAGG
GAGATTTTCTTAAAACAAAACAAAACAAACAAAACAAAACAAAAGGCCTGCAGTAAAATG
CTGGTTTTAGTTATAAATTATTTACTCACTGTCCTAGCGATAAGTTCCTTAAACTCTTTT
ATTATCTCAGCTTTCTTCTTTCTGCAAGGGAGCTGATATAATATATTGCAAAGACTTTCT
ATCACCCTTAATACTTACTGAGGAAAAATTGTGCATCTGAGAGACCTAAAGCTGCATATG
CTTTTATGATTTTTTTATTGAAAAATAAATCATACCATTTGTAAAGAAGAAAAGGAAAGG
AACGAATTGTAGCCTGCCTAGTCACTGATTTGAAAGTGATGCTAGATTGTGTTCTGTAAC
TTTCTGGGCCAGAGCTACCTCTTCTGCAAAACTAAAGGTTTGTCAAAGAAGAGTGAAACA
ATAGATTTAATAGTGGCTTGAAACAAAGAAAACATTTGGAAAATTATTTCCACATAATCA
AATCAGTCATATCAATAGCAAATGATGGTGCTTTTATAAGGTATGGGATTTTGGAAATTC
TACTGCCAAATGAAGATGGACTGCAATTGTAAAACAAAATTGAGGAAAAACAAATTTATT
TTGGGTGCATTGGTTAGTATATTGTCTTTTTTATCACCACACTCATTGCCTTTAATAAGG
GAAAGGCTCAACCTACCATAAAGGTGTTACTGACTATAAACATCACCATTCAGTTTTATG
TCCTGTTCTAACATTTTTTCTGCCTGGCTTATTAATTTTTTTAAATACAGAAGTTTATGG
TTGTTTTGAAAATGTTTTTACTATATATTTGGTTTAGTCATTTCAACTCATAAAGACATA
AGCAAGCAGGCAGATAGACAGACACGTCTGTATGTGGTGACACTCATTGTGTTAATAAAT
CATGAATTCATCATCAGCCATTAAGAAACATTTCAATCTTCCATCTAGAGTTTTTGCCTC
TTGCCTGGTCAGACAGGGAACAGTATATATTCAGGATACAGAAGAAGGACTGGGAAGTTT
CTCAAAAGGAAGAAATCTGATGTGTTTTAAGATAATATTTATATTACATTCATTAACTTC
ATTCTATTTCCCTACAGGTTTAAATGTTTCTTTGGCCACTTGACTACTGAACACAAAATG
AATAACTCAACAAACTCCTCGAACAATGGCTTGGCTATTACCAGTCCTTACAAGACATTT
GAAGTGGTATTTATTGTCCTTGTGGCTGGATCCCTCAGTCTGGTGACCATCATTGGGAAC
ATTCTGGTCATGGTTTCCATTAAAGTCAACCGCCACCTTCAGACTGTCAACAATTACTTC
TTGTTCAGCCTGGCCTGTGCTGACCTCATCATAGGTGTTTTCTCCATGAACTTGTATACC
CTCTACACTGTGATTGGTTACTGGCCTTTGGGACCTGTAGTATGTGACCTTTGGCTAGCA
TTGGACTATGTTGTCAGCAATGCCTCCGTTATGAATCTCCTCATCATCAGCTTTGATAGA
TACTTCTGTGTCACGAAACCTCTGACCTACCCAGTTAAGCGGACCACAAAAATGGCAGGC
ATGATGATTGCAGCTGCGTGGGTCCTTTCCTTCATCCTCTGGGCCCCAGCCATTCTCTTC
TGGCAGTTCATCGTAGGGGTGAGGACTGTGGAGGATGGGGAGTGCTATATTCAGTTCTTT
TCCAATGCGGCCGTCACCTTCGGCACTGCCATTGCAGCTTTCTATCTGCCTGTCATCATC
ATGACTGTGCTCTATTGGCATATATCCCGGGCAAGCAAGAGTAGAATAAAGAAGGAAAAG
AAGGAACCTGTGGCCAACCAAGACCCAGTATCTCCAAGTCTGGTGCAAGGAAGAATTGTA
AAGCCAAACAATAACAATATGCCTGGTGGTGATGGCGGCCTGGAACACAACAAGATCCAG
AATGGCAAGGCTCCACGGGACGGCGTGACTGAAAACTGTGTTCAGGGGGAGGAGAAAGAG
AGCTCCAATGATTCGACGTCAGTCAGTGCTGTGGCCTCCAATATGAGAGATGATGAGATA
ACCCAGGATGAAAACACAGTTTCCACTTCGCTGGGCCACTCCAGAGATGACAACTCTAAG
CAAACATGCATCAAAATTGTCACCAAGGCCCAAAAGGGTGATGTGTGCACCCCAACGAGT
ACCACTGTAGAACTAGTTGGGTCGTCGGGTCAGAATGGGGATGAAAAGCAGAACATTGTA
GCCCGCAAAATCGTGAAGATGACCAAGCAGCCTGCCAAAAAGAAGCCTCCACCATCCCGG
GAAAAGAAAGTGACCAGGACAATCTTGGCTATCCTGTTGGCTTTCATCATAACGTGGGCG
CCATACAATGTCATGGTGCTCATCAATACTTTCTGTGCACCCTGCATCCCCAATACAGTA
TGGACAATTGGCTACTGGCTCTGTTACATCAATAGCACCATCAATCCGGCCTGCTATGCG
CTTTGTAATGCCACCTTCAAAAAGACTTTTAAGCACCTCCTCATGTGTCATTACAAGAAC
ATAGGCGCTACACGGTGAAAAGACCATCAAAAGAAGAAATGTGGTCGAGTGTGTCTTGGG
GAAGAACAGAGACAAGAAAGCTGTGTTTATAGTGACCTGCCATTTCACTTTACAGTCTTA
CTGCAACATGAAAGTAAGGAGTTTTAGAGAGACACTATCATTGTGCCCATGCTCCATTTT
GGGAAAAATAAATTAATAAACCTTCACCTTATAAACCCTGTCAGTTTAGGAGCACCGAGA
AAATGAAAGAGGCATGCTGAAACTGCAGATCTAAGGAAAAATCTCTACTGTCTCCTGCTC
TCTTGAAGAAGGGCGTCAGAGTCTACAATTTCATGTCTCTGCACAAGAAGAATAACCTAG
TCTATTTGTTGTTTCTTTCTGTTGTTCCCCTGTGTGGCGGTGAGAAAGAAATGACACATT
CCATGCTAACACAGAGACCTACATGGAAAGAAGCAGGCACTGTACAATGAGAGAGAAGAA
GAAAGAAAATCAAATAGGATGCAGAGATGGTCTGCAGAGCAGCGAGCATATCCTCGGCTG
TGCTGTGCTCTGATCTGAAGGATTTCAACACAACAACTGCTTATTTCTTCTCTCTTCCCC
TTTCCCGTCATGGAAAGCAAGAAAGCAAAACAACAAAAGCCCAACTAGGCCATACTATTT
TTTTCTACTATTTTTGTAGTTTCTGGTTTTATATTATATATGGATAGTATCTGCAGGACT
TCTAAATCTGGCCAGAGTCACCCATGAAAATGGACACTCTGCCACTCCCCACTGTGTTGT
TGCATTCTTTGATGGTGTACAGACCTGTAAGGATTTCATTCACTTGAGTTCCTCTGTGTG
GGAAATTTTTACCCACCTGATTTTCTTCTCAACAAACCCATTTTAACAATGTTGTCTGCC
TAAGACATAAATACGCTTGGGAAACCAAAGTCATTTAAACTTAATTTTTAATCACATTAG
AAAATGGTCAGTCTAGTTGGAGAGAGTTCACCGAGGCCAGACGAAATTTGAGATGAGAAG
ACAGAAGGTCAGAAGAGCGATCTGTTTGAACTTGAACCTGCCTGACCTATGTGCACAATG
TTAACGTGTACAACATGAACTCCTGACATTGTCAGCAGGAATATGATGTTGCTTTGCTCA
TTTGAGTCTCAATTAGTTCACTGTGACATTACTATCCAATGTCTGTTAGCAGATGGTACT
CAAATTATACTCAAATGAAGTTTTCTATAATCATATTAAGACACAGGGGTGGGGAAATGG
GTCATGCAAAAATATTTACTAATACTACCTGTTCCATGTTCTTCCAGTTGTCTTCAAATC
TCTGTAAGATTATATTGCTTCAAAATTCTTTGTGGCTTCAACATAGTGGATAACCAAAAA
GAGGCAAAATATGCCCACAGAACCTGATGGAAGAGTACATGTGATTCTCGCCATGCCATT
TCCCACAGACTAATCTAACCACGAATAGCTGTCACAGAAAGGATTGTTAAACGGTAACAC
TAAACACAAAAGGTCAATGTAGAAAAATCCATACTGGACACTCTAAAATAATGCGGAATC
ATACCAATCTACATTAACTAGTTATTTTTGTCTTCACATAATTTCTTCTATTATACATTA
CAGTATTATGGTCAGATTTTTCCAAGTCTGGGCCTAATGGATAGAAATTGAAGTTCTAAA
TTCTTCTATAAAGTTCAGATATTCGCTGATGCACTTTGATTCCCTCTCTGCCATTCCACG
AGTATCACAAGGCAGGTTCGTCAGCGTAAAGTCCTAAGAGTTATCAGAACCATTTTGTCT
GACTGCATGGCAGTAACGTCTCAGCAAGTGGGCGATAAAGGATAGAACGGCAGCAACAGC
AACATTGTAAGCTAAAAAGAAACAAGTTGCTAATAATATATTTAACCTTTATGAACAGAA
CTTATTGTTTGAGCAATTTCAGTCGGTTTTAACCTCAAAAACTCCTATCTATAAGACATA
GAATCGGTTCAGATTAGTTTAGTCTATAATTAGTTGACTGAGTTCCCTTAGTTCAGAATT
ACTAAAATGTAAATTCACTTTATGTTGTTTCTTCGAGTAATTGAGTGGAGAAGACTTCGA
TCCAATGGATTTCTTGGTTTCCTGTAGGAATGTTAACAATCTGTGTTATACACTATTAGA
TGCAGACGGGCTTCCCTTTCCAGCACAC
```

[Back to the top]

**D. Pairwise alignment between the original *chrm2* (Query) sequence and the above contig (Sbjct)**

```
 Score = 4107 bits (2072), Expect = 0.0
 Identities = 2072/2072 (100%)
 Strand = Plus / Plus

                                                                        
Query: 1    ccctacaggtttaaatgtttctttggccacttgactactgaacacaaaatgaataactca 60
            ||||||||||||||||||||||||||||||||||||||||||||||||||||||||||||
Sbjct: 1450 ccctacaggtttaaatgtttctttggccacttgactactgaacacaaaatgaataactca 1509

                                                                        
Query: 61   acaaactcctcgaacaatggcttggctattaccagtccttacaagacatttgaagtggta 120
            ||||||||||||||||||||||||||||||||||||||||||||||||||||||||||||
Sbjct: 1510 acaaactcctcgaacaatggcttggctattaccagtccttacaagacatttgaagtggta 1569

                                                                        
Query: 121  tttattgtccttgtggctggatccctcagtctggtgaccatcattgggaacattctggtc 180
            ||||||||||||||||||||||||||||||||||||||||||||||||||||||||||||
Sbjct: 1570 tttattgtccttgtggctggatccctcagtctggtgaccatcattgggaacattctggtc 1629

                                                                        
Query: 181  atggtttccattaaagtcaaccgccaccttcagactgtcaacaattacttcttgttcagc 240
            ||||||||||||||||||||||||||||||||||||||||||||||||||||||||||||
Sbjct: 1630 atggtttccattaaagtcaaccgccaccttcagactgtcaacaattacttcttgttcagc 1689

                                                                        
Query: 241  ctggcctgtgctgacctcatcataggtgttttctccatgaacttgtataccctctacact 300
            ||||||||||||||||||||||||||||||||||||||||||||||||||||||||||||
Sbjct: 1690 ctggcctgtgctgacctcatcataggtgttttctccatgaacttgtataccctctacact 1749

                                                                        
Query: 301  gtgattggttactggcctttgggacctgtagtatgtgacctttggctagcattggactat 360
            ||||||||||||||||||||||||||||||||||||||||||||||||||||||||||||
Sbjct: 1750 gtgattggttactggcctttgggacctgtagtatgtgacctttggctagcattggactat 1809

                                                                        
Query: 361  gttgtcagcaatgcctccgttatgaatctcctcatcatcagctttgatagatacttctgt 420
            ||||||||||||||||||||||||||||||||||||||||||||||||||||||||||||
Sbjct: 1810 gttgtcagcaatgcctccgttatgaatctcctcatcatcagctttgatagatacttctgt 1869

                                                                        
Query: 421  gtcacgaaacctctgacctacccagttaagcggaccacaaaaatggcaggcatgatgatt 480
            ||||||||||||||||||||||||||||||||||||||||||||||||||||||||||||
Sbjct: 1870 gtcacgaaacctctgacctacccagttaagcggaccacaaaaatggcaggcatgatgatt 1929

                                                                        
Query: 481  gcagctgcgtgggtcctttccttcatcctctgggccccagccattctcttctggcagttc 540
            ||||||||||||||||||||||||||||||||||||||||||||||||||||||||||||
Sbjct: 1930 gcagctgcgtgggtcctttccttcatcctctgggccccagccattctcttctggcagttc 1989

                                                                        
Query: 541  atcgtaggggtgaggactgtggaggatggggagtgctatattcagttcttttccaatgcg 600
            ||||||||||||||||||||||||||||||||||||||||||||||||||||||||||||
Sbjct: 1990 atcgtaggggtgaggactgtggaggatggggagtgctatattcagttcttttccaatgcg 2049

                                                                        
Query: 601  gccgtcaccttcggcactgccattgcagctttctatctgcctgtcatcatcatgactgtg 660
            ||||||||||||||||||||||||||||||||||||||||||||||||||||||||||||
Sbjct: 2050 gccgtcaccttcggcactgccattgcagctttctatctgcctgtcatcatcatgactgtg 2109

                                                                        
Query: 661  ctctattggcatatatcccgggcaagcaagagtagaataaagaaggaaaagaaggaacct 720
            ||||||||||||||||||||||||||||||||||||||||||||||||||||||||||||
Sbjct: 2110 ctctattggcatatatcccgggcaagcaagagtagaataaagaaggaaaagaaggaacct 2169

                                                                        
Query: 721  gtggccaaccaagacccagtatctccaagtctggtgcaaggaagaattgtaaagccaaac 780
            ||||||||||||||||||||||||||||||||||||||||||||||||||||||||||||
Sbjct: 2170 gtggccaaccaagacccagtatctccaagtctggtgcaaggaagaattgtaaagccaaac 2229

                                                                        
Query: 781  aataacaatatgcctggtggtgatggcggcctggaacacaacaagatccagaatggcaag 840
            ||||||||||||||||||||||||||||||||||||||||||||||||||||||||||||
Sbjct: 2230 aataacaatatgcctggtggtgatggcggcctggaacacaacaagatccagaatggcaag 2289

                                                                        
Query: 841  gctccacgggacggcgtgactgaaaactgtgttcagggggaggagaaagagagctccaat 900
            ||||||||||||||||||||||||||||||||||||||||||||||||||||||||||||
Sbjct: 2290 gctccacgggacggcgtgactgaaaactgtgttcagggggaggagaaagagagctccaat 2349

                                                                        
Query: 901  gattcgacgtcagtcagtgctgtggcctccaatatgagagatgatgagataacccaggat 960
            ||||||||||||||||||||||||||||||||||||||||||||||||||||||||||||
Sbjct: 2350 gattcgacgtcagtcagtgctgtggcctccaatatgagagatgatgagataacccaggat 2409

                                                                        
Query: 961  gaaaacacagtttccacttcgctgggccactccagagatgacaactctaagcaaacatgc 1020
            ||||||||||||||||||||||||||||||||||||||||||||||||||||||||||||
Sbjct: 2410 gaaaacacagtttccacttcgctgggccactccagagatgacaactctaagcaaacatgc 2469

                                                                        
Query: 1021 atcaaaattgtcaccaaggcccaaaagggtgatgtgtgcaccccaacgagtaccactgta 1080
            ||||||||||||||||||||||||||||||||||||||||||||||||||||||||||||
Sbjct: 2470 atcaaaattgtcaccaaggcccaaaagggtgatgtgtgcaccccaacgagtaccactgta 2529

                                                                        
Query: 1081 gaactagttgggtcgtcgggtcagaatggggatgaaaagcagaacattgtagcccgcaaa 1140
            ||||||||||||||||||||||||||||||||||||||||||||||||||||||||||||
Sbjct: 2530 gaactagttgggtcgtcgggtcagaatggggatgaaaagcagaacattgtagcccgcaaa 2589

                                                                        
Query: 1141 atcgtgaagatgaccaagcagcctgccaaaaagaagcctccaccatcccgggaaaagaaa 1200
            ||||||||||||||||||||||||||||||||||||||||||||||||||||||||||||
Sbjct: 2590 atcgtgaagatgaccaagcagcctgccaaaaagaagcctccaccatcccgggaaaagaaa 2649

                                                                        
Query: 1201 gtgaccaggacaatcttggctatcctgttggctttcatcataacgtgggcgccatacaat 1260
            ||||||||||||||||||||||||||||||||||||||||||||||||||||||||||||
Sbjct: 2650 gtgaccaggacaatcttggctatcctgttggctttcatcataacgtgggcgccatacaat 2709

                                                                        
Query: 1261 gtcatggtgctcatcaatactttctgtgcaccctgcatccccaatacagtatggacaatt 1320
            ||||||||||||||||||||||||||||||||||||||||||||||||||||||||||||
Sbjct: 2710 gtcatggtgctcatcaatactttctgtgcaccctgcatccccaatacagtatggacaatt 2769

                                                                        
Query: 1321 ggctactggctctgttacatcaatagcaccatcaatccggcctgctatgcgctttgtaat 1380
            ||||||||||||||||||||||||||||||||||||||||||||||||||||||||||||
Sbjct: 2770 ggctactggctctgttacatcaatagcaccatcaatccggcctgctatgcgctttgtaat 2829

                                                                        
Query: 1381 gccaccttcaaaaagacttttaagcacctcctcatgtgtcattacaagaacataggcgct 1440
            ||||||||||||||||||||||||||||||||||||||||||||||||||||||||||||
Sbjct: 2830 gccaccttcaaaaagacttttaagcacctcctcatgtgtcattacaagaacataggcgct 2889

                                                                        
Query: 1441 acacggtgaaaagaccatcaaaagaagaaatgtggtcgagtgtgtcttggggaagaacag 1500
            ||||||||||||||||||||||||||||||||||||||||||||||||||||||||||||
Sbjct: 2890 acacggtgaaaagaccatcaaaagaagaaatgtggtcgagtgtgtcttggggaagaacag 2949

                                                                        
Query: 1501 agacaagaaagctgtgtttatagtgacctgccatttcactttacagtcttactgcaacat 1560
            ||||||||||||||||||||||||||||||||||||||||||||||||||||||||||||
Sbjct: 2950 agacaagaaagctgtgtttatagtgacctgccatttcactttacagtcttactgcaacat 3009

                                                                        
Query: 1561 gaaagtaaggagttttagagagacactatcattgtgcccatgctccattttgggaaaaat 1620
            ||||||||||||||||||||||||||||||||||||||||||||||||||||||||||||
Sbjct: 3010 gaaagtaaggagttttagagagacactatcattgtgcccatgctccattttgggaaaaat 3069

                                                                        
Query: 1621 aaattaataaaccttcaccttataaaccctgtcagtttaggagcaccgagaaaatgaaag 1680
            ||||||||||||||||||||||||||||||||||||||||||||||||||||||||||||
Sbjct: 3070 aaattaataaaccttcaccttataaaccctgtcagtttaggagcaccgagaaaatgaaag 3129

                                                                        
Query: 1681 aggcatgctgaaactgcagatctaaggaaaaatctctactgtctcctgctctcttgaaga 1740
            ||||||||||||||||||||||||||||||||||||||||||||||||||||||||||||
Sbjct: 3130 aggcatgctgaaactgcagatctaaggaaaaatctctactgtctcctgctctcttgaaga 3189

                                                                        
Query: 1741 agggcgtcagagtctacaatttcatgtctctgcacaagaagaataacctagtctatttgt 1800
            ||||||||||||||||||||||||||||||||||||||||||||||||||||||||||||
Sbjct: 3190 agggcgtcagagtctacaatttcatgtctctgcacaagaagaataacctagtctatttgt 3249

                                                                        
Query: 1801 tgtttctttctgttgttcccctgtgtggcggtgagaaagaaatgacacattccatgctaa 1860
            ||||||||||||||||||||||||||||||||||||||||||||||||||||||||||||
Sbjct: 3250 tgtttctttctgttgttcccctgtgtggcggtgagaaagaaatgacacattccatgctaa 3309

                                                                        
Query: 1861 cacagagacctacatggaaagaagcaggcactgtacaatgagagagaagaagaaagaaaa 1920
            ||||||||||||||||||||||||||||||||||||||||||||||||||||||||||||
Sbjct: 3310 cacagagacctacatggaaagaagcaggcactgtacaatgagagagaagaagaaagaaaa 3369

                                                                        
Query: 1921 tcaaataggatgcagagatggtctgcagagcagcgagcatatcctcggctgtgctgtgct 1980
            ||||||||||||||||||||||||||||||||||||||||||||||||||||||||||||
Sbjct: 3370 tcaaataggatgcagagatggtctgcagagcagcgagcatatcctcggctgtgctgtgct 3429

                                                                        
Query: 1981 ctgatctgaaggatttcaacacaacaactgcttatttcttctctcttcccctttcccgtc 2040
            ||||||||||||||||||||||||||||||||||||||||||||||||||||||||||||
Sbjct: 3430 ctgatctgaaggatttcaacacaacaactgcttatttcttctctcttcccctttcccgtc 3489

                                            
Query: 2041 atggaaagcaagaaagcaaaacaacaaaagcc 2072
            ||||||||||||||||||||||||||||||||
Sbjct: 3490 atggaaagcaagaaagcaaaacaacaaaagcc 3521
```

[Back to the top]

**E. Pairwise alignment between the above contig (Query) and entire rat Chromosome 4 (Sbjct), which is gi|62750804|ref|NC\_005103.2|NC\_005103Rattus norvegicus chromosome 4, reference assembly (based on RGSC v3.4).**

The alignment was performed with the BLAST2 web server at NCBI (http://www.ncbi.nlm.nih.gov/blast/bl2seq/wblast2.cgi) with its 'Filter' option un-checked.

```
 Score = 9662 bits (5025),  Expect = 0.0
 Identities = 5045/5050 (99%), Gaps = 2/5050 (0%)
 Strand=Plus/Plus

Query  1         AAGCTGGTGGAAGCCAGCTACTATCTCTCATAAAAAAGGAACAGCCTTCACATAATGTGT  60
                 |||||||||||||||||||||||||||||||||||||||||| |||||||||||||||||
Sbjct  63909839  AAGCTGGTGGAAGCCAGCTACTATCTCTCATAAAAAAGGAACGGCCTTCACATAATGTGT  63909898

Query  61        GCATCCCCTTGGAAGATGGGATGTCCCTGCTTCGAATACTTGCGCTTGCTTCTAAATGGC  120
                 || ||||||||||||||||||| |||||||||||||||||||||||||||||||||||||
Sbjct  63909899  GCCTCCCCTTGGAAGATGGGATATCCCTGCTTCGAATACTTGCGCTTGCTTCTAAATGGC  63909958

Query  121       ATGTTTCCCATTGTCACACCAGTACTGCCACAGACCAGTAGGGCGTCTCTAAAGATTCTG  180
                 ||||||||||||||||||||||||||||||||||||||||||||||||||||||||||||
Sbjct  63909959  ATGTTTCCCATTGTCACACCAGTACTGCCACAGACCAGTAGGGCGTCTCTAAAGATTCTG  63910018

Query  181       AGCTGCAATAGTCAATATAGTTCCCGTCCCATAGAGTTTTCAGTGTTCAATATGGATAAG  240
                 ||||||||||||||||||||||||||||||||||||||||||||||||||||||||||||
Sbjct  63910019  AGCTGCAATAGTCAATATAGTTCCCGTCCCATAGAGTTTTCAGTGTTCAATATGGATAAG  63910078

Query  241       GATCCCAGGTGCATAAACTCTCCGTTTTGACTAGCAACGTCTGCACATAAGGCATCATCA  300
                 ||||||||||||||||||||||||||||||||||||||||||||||||||||||||||||
Sbjct  63910079  GATCCCAGGTGCATAAACTCTCCGTTTTGACTAGCAACGTCTGCACATAAGGCATCATCA  63910138

Query  301       TGTTGCTCCTCGGGAATCTCTCATTAAGTGCAACCCTTCAATGGAAAAGGGAGGCAGAGG  360
                 ||||||||||||||||||||||||||||||||||||||||||||||||||||||||||||
Sbjct  63910139  TGTTGCTCCTCGGGAATCTCTCATTAAGTGCAACCCTTCAATGGAAAAGGGAGGCAGAGG  63910198

Query  361       GAGATTTTCTTAAAACAAAACAAAACAAACAAAACAAAACAAAAGGCCTGCAGTAAAATG  420
                 ||||||||||||||||||||||||||||||||||||||||||||||||||||||||||||
Sbjct  63910199  GAGATTTTCTTAAAACAAAACAAAACAAACAAAACAAAACAAAAGGCCTGCAGTAAAATG  63910258

Query  421       CTGGTTTTAGTTATAAATTATTTACTCACTGTCCTAGCGATAAGTTCCTTAAACTCTTTT  480
                 ||||||||||||||||||||||||||||||||||||||||||||||||||||||||||||
Sbjct  63910259  CTGGTTTTAGTTATAAATTATTTACTCACTGTCCTAGCGATAAGTTCCTTAAACTCTTTT  63910318

Query  481       ATTATCTCAGCTTTCTTCTTTCTGCAAGGGAGCTGATATAATATATTGCAAAGACTTTCT  540
                 ||||||||||||||||||||||||||||||||||||||||||||||||||||||||||||
Sbjct  63910319  ATTATCTCAGCTTTCTTCTTTCTGCAAGGGAGCTGATATAATATATTGCAAAGACTTTCT  63910378

Query  541       ATCACCCTTAATACTTACTGAGGAAAAATTGTGCATCTGAGAGACCTAAAGCTGCATATG  600
                 ||||||||||||||||||||||||||||||||||||||||||||||||||||||||||||
Sbjct  63910379  ATCACCCTTAATACTTACTGAGGAAAAATTGTGCATCTGAGAGACCTAAAGCTGCATATG  63910438

Query  601       CTTTTATGATTTTTTTATTGAAAAATAAATCATACCATTTGTAAAGAAGAAAAGGAAAGG  660
                 ||||||||||||||||||||||||||||||||||||||||||||||||||||||||||||
Sbjct  63910439  CTTTTATGATTTTTTTATTGAAAAATAAATCATACCATTTGTAAAGAAGAAAAGGAAAGG  63910498

Query  661       AACGAATTGTAGCCTGCCTAGTCACTGATTTGAAAGTGATGCTAGATTGTGTTCTGTAAC  720
                 ||||||||||||||||||||||||||||||||||||||||||||||||||||||||||||
Sbjct  63910499  AACGAATTGTAGCCTGCCTAGTCACTGATTTGAAAGTGATGCTAGATTGTGTTCTGTAAC  63910558

Query  721       TTTCTGGGCCAGAGCTACCTCTTCTGCAAAACTAAAGGTTTGTCAAAGAAGAGTGAAACA  780
                 ||||||||||||||||||||||||||||||||||||||||||||||||||||||||||||
Sbjct  63910559  TTTCTGGGCCAGAGCTACCTCTTCTGCAAAACTAAAGGTTTGTCAAAGAAGAGTGAAACA  63910618

Query  781       ATAGATTTAATAGTGGCTTGAAACAAAGAAAACATTTGGAAAATTATTTCCACATAATCA  840
                 ||||||||||||||||||||||||||||||||||||||||||||||||||||||||||||
Sbjct  63910619  ATAGATTTAATAGTGGCTTGAAACAAAGAAAACATTTGGAAAATTATTTCCACATAATCA  63910678

Query  841       AATCAGTCATATCAATAGCAAATGATGGTGCTTTTATAAGGTATGGGATTTTGGAAATTC  900
                 ||||||||||||||||||||||||||||||||||||||||||||||||||||||||||||
Sbjct  63910679  AATCAGTCATATCAATAGCAAATGATGGTGCTTTTATAAGGTATGGGATTTTGGAAATTC  63910738

Query  901       TACTGCCAAATGAAGATGGACTGCAATTGTAAAACAAAATTGAGGAAAAACAAATTTATT  960
                 ||||||||||||||||||||||||||||||||||||||||||||||||||||||||||||
Sbjct  63910739  TACTGCCAAATGAAGATGGACTGCAATTGTAAAACAAAATTGAGGAAAAACAAATTTATT  63910798

Query  961       TTGGGTGCATTGGTTAGTATATTGTCTTTTTTATCACCACACTCATTGCCTTTAATAAGG  1020
                 ||||||||||||||||||||||||||||||||||||||||||||||||||||||||||||
Sbjct  63910799  TTGGGTGCATTGGTTAGTATATTGTCTTTTTTATCACCACACTCATTGCCTTTAATAAGG  63910858

Query  1021      GAAAGGCTCAACCTACCATAAAGGTGTTACTGACTATAAACATCACCATTCAGTTTTATG  1080
                 ||||||||||||||||||||||||||||||||||||||||||||||||||||||||||||
Sbjct  63910859  GAAAGGCTCAACCTACCATAAAGGTGTTACTGACTATAAACATCACCATTCAGTTTTATG  63910918

Query  1081      TCCTGTTCTAACATTTTTTCTGCCTGGCTTATTAATTTTTTTAAATACAGAAGTTTATGG  1140
                 ||||||||||||||||||||||||||||||||||||||||||||||||||||||||||||
Sbjct  63910919  TCCTGTTCTAACATTTTTTCTGCCTGGCTTATTAATTTTTTTAAATACAGAAGTTTATGG  63910978

Query  1141      TTGTTTTGAAAATGTTTTTACTATATATTTGGTTTAGTCATTTCAACTCATAAAGACATA  1200
                 ||||||||||||||||||||||||||||||||||||||||||||||||||||||||||||
Sbjct  63910979  TTGTTTTGAAAATGTTTTTACTATATATTTGGTTTAGTCATTTCAACTCATAAAGACATA  63911038

Query  1201      AGCAAGCAGGCAGATAGACAGACACGTCTGTATGTGGTGACACTCATTGTGTTAATAAAT  1260
                 ||||||||||||||||||||||||||||||||||||||||||||||||||||||||||||
Sbjct  63911039  AGCAAGCAGGCAGATAGACAGACACGTCTGTATGTGGTGACACTCATTGTGTTAATAAAT  63911098

Query  1261      CATGAATTCATCATCAGCCATTAAGAAACATTTCAATCTTCCATCTAGAGTTTTTGCCTC  1320
                 ||||||||||||||||||||||||||||||||||||||||||||||||||||||||||||
Sbjct  63911099  CATGAATTCATCATCAGCCATTAAGAAACATTTCAATCTTCCATCTAGAGTTTTTGCCTC  63911158

Query  1321      TTGCCTGGTCAGACAGGGAACAGTATATATTCAGGATACAGAAGAAGGACTGGGAAGTTT  1380
                 ||||||||||||||||||||||||||||||||||||||||||||||||||||||||||||
Sbjct  63911159  TTGCCTGGTCAGACAGGGAACAGTATATATTCAGGATACAGAAGAAGGACTGGGAAGTTT  63911218

Query  1381      CTCAAAAGGAAGAAATCTGATGTGTTTTAAGATAATATTTATATTACATTCATTAACTTC  1440
                 ||||||||||||||||||||||||||||||||||||||||||||||||||||||||||||
Sbjct  63911219  CTCAAAAGGAAGAAATCTGATGTGTTTTAAGATAATATTTATATTACATTCATTAACTTC  63911278

Query  1441      ATTCTATTTCCCTACAGGTTTAAATGTTTCTTTGGCCACTTGACTACTGAACACAAAATG  1500
                 ||||||||||||||||||||||||||||||||||||||||||||||||||||||||||||
Sbjct  63911279  ATTCTATTTCCCTACAGGTTTAAATGTTTCTTTGGCCACTTGACTACTGAACACAAAATG  63911338

Query  1501      AATAACTCAACAAACTCCTCGAACAATGGCTTGGCTATTACCAGTCCTTACAAGACATTT  1560
                 ||||||||||||||||||||||||||||||||||||||||||||||||||||||||||||
Sbjct  63911339  AATAACTCAACAAACTCCTCGAACAATGGCTTGGCTATTACCAGTCCTTACAAGACATTT  63911398

Query  1561      GAAGTGGTATTTATTGTCCTTGTGGCTGGATCCCTCAGTCTGGTGACCATCATTGGGAAC  1620
                 ||||||||||||||||||||||||||||||||||||||||||||||||||||||||||||
Sbjct  63911399  GAAGTGGTATTTATTGTCCTTGTGGCTGGATCCCTCAGTCTGGTGACCATCATTGGGAAC  63911458

Query  1621      ATTCTGGTCATGGTTTCCATTAAAGTCAACCGCCACCTTCAGACTGTCAACAATTACTTC  1680
                 ||||||||||||||||||||||||||||||||||||||||||||||||||||||||||||
Sbjct  63911459  ATTCTGGTCATGGTTTCCATTAAAGTCAACCGCCACCTTCAGACTGTCAACAATTACTTC  63911518

Query  1681      TTGTTCAGCCTGGCCTGTGCTGACCTCATCATAGGTGTTTTCTCCATGAACTTGTATACC  1740
                 ||||||||||||||||||||||||||||||||||||||||||||||||||||||||||||
Sbjct  63911519  TTGTTCAGCCTGGCCTGTGCTGACCTCATCATAGGTGTTTTCTCCATGAACTTGTATACC  63911578

Query  1741      CTCTACACTGTGATTGGTTACTGGCCTTTGGGACCTGTAGTATGTGACCTTTGGCTAGCA  1800
                 ||||||||||||||||||||||||||||||||||||||||||||||||||||||||||||
Sbjct  63911579  CTCTACACTGTGATTGGTTACTGGCCTTTGGGACCTGTAGTATGTGACCTTTGGCTAGCA  63911638

Query  1801      TTGGACTATGTTGTCAGCAATGCCTCCGTTATGAATCTCCTCATCATCAGCTTTGATAGA  1860
                 ||||||||||||||||||||||||||||||||||||||||||||||||||||||||||||
Sbjct  63911639  TTGGACTATGTTGTCAGCAATGCCTCCGTTATGAATCTCCTCATCATCAGCTTTGATAGA  63911698

Query  1861      TACTTCTGTGTCACGAAACCTCTGACCTACCCAGTTAAGCGGACCACAAAAATGGCAGGC  1920
                 ||||||||||||||||||||||||||||||||||||||||||||||||||||||||||||
Sbjct  63911699  TACTTCTGTGTCACGAAACCTCTGACCTACCCAGTTAAGCGGACCACAAAAATGGCAGGC  63911758

Query  1921      ATGATGATTGCAGCTGCGTGGGTCCTTTCCTTCATCCTCTGGGCCCCAGCCATTCTCTTC  1980
                 ||||||||||||||||||||||||||||||||||||||||||||||||||||||||||||
Sbjct  63911759  ATGATGATTGCAGCTGCGTGGGTCCTTTCCTTCATCCTCTGGGCCCCAGCCATTCTCTTC  63911818

Query  1981      TGGCAGTTCATCGTAGGGGTGAGGACTGTGGAGGATGGGGAGTGCTATATTCAGTTCTTT  2040
                 ||||||||||||||||||||||||||||||||||||||||||||||||||||||||||||
Sbjct  63911819  TGGCAGTTCATCGTAGGGGTGAGGACTGTGGAGGATGGGGAGTGCTATATTCAGTTCTTT  63911878

Query  2041      TCCAATGCGGCCGTCACCTTCGGCACTGCCATTGCAGCTTTCTATCTGCCTGTCATCATC  2100
                 ||||||||||||||||||||||||||||||||||||||||||||||||||||||||||||
Sbjct  63911879  TCCAATGCGGCCGTCACCTTCGGCACTGCCATTGCAGCTTTCTATCTGCCTGTCATCATC  63911938

Query  2101      ATGACTGTGCTCTATTGGCATATATCCCGGGCAAGCAAGAGTAGAATAAAGAAGGAAAAG  2160
                 ||||||||||||||||||||||||||||||||||||||||||||||||||||||||||||
Sbjct  63911939  ATGACTGTGCTCTATTGGCATATATCCCGGGCAAGCAAGAGTAGAATAAAGAAGGAAAAG  63911998

Query  2161      AAGGAACCTGTGGCCAACCAAGACCCAGTATCTCCAAGTCTGGTGCAAGGAAGAATTGTA  2220
                 ||||||||||||||||||||||||||||||||||||||||||||||||||||||||||||
Sbjct  63911999  AAGGAACCTGTGGCCAACCAAGACCCAGTATCTCCAAGTCTGGTGCAAGGAAGAATTGTA  63912058

Query  2221      AAGCCAAACAATAACAATATGCCTGGTGGTGATGGCGGCCTGGAACACAACAAGATCCAG  2280
                 ||||||||||||||||||||||||||||||||||||||||||||||||||||||||||||
Sbjct  63912059  AAGCCAAACAATAACAATATGCCTGGTGGTGATGGCGGCCTGGAACACAACAAGATCCAG  63912118

Query  2281      AATGGCAAGGCTCCACGGGACGGCGTGACTGAAAACTGTGTTCAGGGGGAGGAGAAAGAG  2340
                 ||||||||||||||||||||||||||||||||||||||||||||||||||||||||||||
Sbjct  63912119  AATGGCAAGGCTCCACGGGACGGCGTGACTGAAAACTGTGTTCAGGGGGAGGAGAAAGAG  63912178

Query  2341      AGCTCCAATGATTCGACGTCAGTCAGTGCTGTGGCCTCCAATATGAGAGATGATGAGATA  2400
                 ||||||||||||||||||||||||||||||||||||||||||||||||||||||||||||
Sbjct  63912179  AGCTCCAATGATTCGACGTCAGTCAGTGCTGTGGCCTCCAATATGAGAGATGATGAGATA  63912238

Query  2401      ACCCAGGATGAAAACACAGTTTCCACTTCGCTGGGCCACTCCAGAGATGACAACTCTAAG  2460
                 ||||||||||||||||||||||||||||||||||||||||||||||||||||||||||||
Sbjct  63912239  ACCCAGGATGAAAACACAGTTTCCACTTCGCTGGGCCACTCCAGAGATGACAACTCTAAG  63912298

Query  2461      CAAACATGCATCAAAATTGTCACCAAGGCCCAAAAGGGTGATGTGTGCACCCCAACGAGT  2520
                 ||||||||||||||||||||||||||||||||||||||||||||||||||||||||||||
Sbjct  63912299  CAAACATGCATCAAAATTGTCACCAAGGCCCAAAAGGGTGATGTGTGCACCCCAACGAGT  63912358

Query  2521      ACCACTGTAGAACTAGTTGGGTCGTCGGGTCAGAATGGGGATGAAAAGCAGAACATTGTA  2580
                 ||||||||||||||||||||||||||||||||||||||||||||||||||||||||||||
Sbjct  63912359  ACCACTGTAGAACTAGTTGGGTCGTCGGGTCAGAATGGGGATGAAAAGCAGAACATTGTA  63912418

Query  2581      GCCCGCAAAATCGTGAAGATGACCAAGCAGCCTGCCAAAAAGAAGCCTCCACCATCCCGG  2640
                 ||||||||||||||||||||||||||||||||||||||||||||||||||||||||||||
Sbjct  63912419  GCCCGCAAAATCGTGAAGATGACCAAGCAGCCTGCCAAAAAGAAGCCTCCACCATCCCGG  63912478

Query  2641      GAAAAGAAAGTGACCAGGACAATCTTGGCTATCCTGTTGGCTTTCATCATAACGTGGGCG  2700
                 ||||||||||||||||||||||||||||||||||||||||||||||||||||||||||||
Sbjct  63912479  GAAAAGAAAGTGACCAGGACAATCTTGGCTATCCTGTTGGCTTTCATCATAACGTGGGCG  63912538

Query  2701      CCATACAATGTCATGGTGCTCATCAATACTTTCTGTGCACCCTGCATCCCCAATACAGTA  2760
                 ||||||||||||||||||||||||||||||||||||||||||||||||||||||||||||
Sbjct  63912539  CCATACAATGTCATGGTGCTCATCAATACTTTCTGTGCACCCTGCATCCCCAATACAGTA  63912598

Query  2761      TGGACAATTGGCTACTGGCTCTGTTACATCAATAGCACCATCAATCCGGCCTGCTATGCG  2820
                 ||||||||||||||||||||||||||||||||||||||||||||||||||||||||||||
Sbjct  63912599  TGGACAATTGGCTACTGGCTCTGTTACATCAATAGCACCATCAATCCGGCCTGCTATGCG  63912658

Query  2821      CTTTGTAATGCCACCTTCAAAAAGACTTTTAAGCACCTCCTCATGTGTCATTACAAGAAC  2880
                 ||||||||||||||||||||||||||||||||||||||||||||||||||||||||||||
Sbjct  63912659  CTTTGTAATGCCACCTTCAAAAAGACTTTTAAGCACCTCCTCATGTGTCATTACAAGAAC  63912718

Query  2881      ATAGGCGCTACACGGTGAAAAGACCATCAAAAGAAGAAATGTGGTCGAGTGTGTCTTGGG  2940
                 ||||||||||||||||||||||||||||||||||||||||||||||||||||||||||||
Sbjct  63912719  ATAGGCGCTACACGGTGAAAAGACCATCAAAAGAAGAAATGTGGTCGAGTGTGTCTTGGG  63912778

Query  2941      GAAGAACAGAGACAAGAAAGCTGTGTTTATAGTGACCTGCCATTTCACTTTACAGTCTTA  3000
                 ||||||||||||||||||||||||||||||||||||||||||||||||||||||||||||
Sbjct  63912779  GAAGAACAGAGACAAGAAAGCTGTGTTTATAGTGACCTGCCATTTCACTTTACAGTCTTA  63912838

Query  3001      CTGCAACATGAAAGTAAGGAGTTTTAGAGAGACACTATCATTGTGCCCATGCTCCATTTT  3060
                 ||||||||||||||||||||||||||||||||||||||||||||||||||||||||||||
Sbjct  63912839  CTGCAACATGAAAGTAAGGAGTTTTAGAGAGACACTATCATTGTGCCCATGCTCCATTTT  63912898

Query  3061      GGGAAAAATAAATTAATAAACCTTCACCTTATAAACCCTGTCAGTTTAGGAGCACCGAGA  3120
                 ||||||||||||||||||||||||||||||||||||||||||||||||||||||||||||
Sbjct  63912899  GGGAAAAATAAATTAATAAACCTTCACCTTATAAACCCTGTCAGTTTAGGAGCACCGAGA  63912958

Query  3121      AAATGAAAGAGGCATGCTGAAACTGCAGATCTAAGGAAAAATCTCTACTGTCTCCTGCTC  3180
                 ||||||||||||||||||||||||||||||||||||||||||||||||||||||||||||
Sbjct  63912959  AAATGAAAGAGGCATGCTGAAACTGCAGATCTAAGGAAAAATCTCTACTGTCTCCTGCTC  63913018

Query  3181      TCTTGAAGAAGGGCGTCAGAGTCTACAATTTCATGTCTCTGCACAAGAAGAATAACCTAG  3240
                 ||||||||||||||||||||||||||||||||||||||||||||||||||||||||||||
Sbjct  63913019  TCTTGAAGAAGGGCGTCAGAGTCTACAATTTCATGTCTCTGCACAAGAAGAATAACCTAG  63913078

Query  3241      TCTATTTGTTGTTTCTTTCTGTTGTTCCCCTGTGTGGCGGTGAGAAAGAAATGACACATT  3300
                 ||||||||||||||||||||||||||||||||||||||||||||||||||||||||||||
Sbjct  63913079  TCTATTTGTTGTTTCTTTCTGTTGTTCCCCTGTGTGGCGGTGAGAAAGAAATGACACATT  63913138

Query  3301      CCATGCTAACACAGAGACCTACATGGAAAGAAGCAGGCACTGTACAATGAGAGAGAAGAA  3360
                 ||||||||||||||||||||||||||||||||||||||||||||||||||||||||||||
Sbjct  63913139  CCATGCTAACACAGAGACCTACATGGAAAGAAGCAGGCACTGTACAATGAGAGAGAAGAA  63913198

Query  3361      GAAAGAAAATCAAATAGGATGCAGAGATGGTCTGCAGAGCAGCGAGCATATCCTCGGCTG  3420
                 ||||||||||||||||||||||||||||||||||||||||||||||||||||||||||||
Sbjct  63913199  GAAAGAAAATCAAATAGGATGCAGAGATGGTCTGCAGAGCAGCGAGCATATCCTCGGCTG  63913258

Query  3421      TGCTGTGCTCTGATCTGAAGGATTTCAACACAACAACTGCTTATTTCTTCTCTCTTCCCC  3480
                 ||||||||||||||||||||||||||||||||||||||||||||||||||||||||||||
Sbjct  63913259  TGCTGTGCTCTGATCTGAAGGATTTCAACACAACAACTGCTTATTTCTTCTCTCTTCCCC  63913318

Query  3481      TTTCCCGTCATGGAAAGCAAGAAAGCAAAACAACAAAAGCCCAACTAGGCCATACTATTT  3540
                 ||||||||||||||||||||||||||||||||||||||||||||||||||||||||||||
Sbjct  63913319  TTTCCCGTCATGGAAAGCAAGAAAGCAAAACAACAAAAGCCCAACTAGGCCATACTATTT  63913378

Query  3541      TTTTCTACTATTTTTGTAGTTTCTGGTTTTATATTATATATGGATAGTATCTGCAGGACT  3600
                 ||||||||||||||||||||||||||||||||||||||||||||||||||||||||||||
Sbjct  63913379  TTTTCTACTATTTTTGTAGTTTCTGGTTTTATATTATATATGGATAGTATCTGCAGGACT  63913438

Query  3601      TCTAAATCTGGCCAGAGTCACCCATGAAAATGGACACTCTGCCACTCCCCACTGTGTTGT  3660
                 ||||||||||||||||||||||||||||||||||||||||||||||||||||||||||||
Sbjct  63913439  TCTAAATCTGGCCAGAGTCACCCATGAAAATGGACACTCTGCCACTCCCCACTGTGTTGT  63913498

Query  3661      TGCATTCTTTGATGGTGTACAGACCTGTAAGGATTTCATTCACTTGAGTTCCTCTGTGTG  3720
                 ||||||||||||||||||||||||||||||||||||||||||||||||||||||||||||
Sbjct  63913499  TGCATTCTTTGATGGTGTACAGACCTGTAAGGATTTCATTCACTTGAGTTCCTCTGTGTG  63913558

Query  3721      GGAAATTTTTACCCACCTGATTTTCTTCTCAACAAACCCATTTTAACAATGTTGTCTGCC  3780
                 ||||||||||||||||||||||||||||||||||||||||||||||||||||||||||||
Sbjct  63913559  GGAAATTTTTACCCACCTGATTTTCTTCTCAACAAACCCATTTTAACAATGTTGTCTGCC  63913618

Query  3781      TAAGACATAAATACGCTTGGGAAACCAAAGTCATTTAAACTTAATTTTTAATCACATTAG  3840
                 ||||||||||||||||||||||||||||||||||||||||||||||||||||||||||||
Sbjct  63913619  TAAGACATAAATACGCTTGGGAAACCAAAGTCATTTAAACTTAATTTTTAATCACATTAG  63913678

Query  3841      AAAATGGTCAGTCTAGTTGGAGAGAGTTCACCGAGGCCAGACGAAATTTGAGATGAGAAG  3900
                 ||||||||||||||||||||||||||||||||||||||||||||||||||||||||||||
Sbjct  63913679  AAAATGGTCAGTCTAGTTGGAGAGAGTTCACCGAGGCCAGACGAAATTTGAGATGAGAAG  63913738

Query  3901      ACAGAAGGTCAGAAGAGCGATCTGTTTGAACTTGAACCTGCCTGACCTATGTGCACAATG  3960
                 ||||||||||||||||||||||||||||||||||||||||||||||||||||||||||||
Sbjct  63913739  ACAGAAGGTCAGAAGAGCGATCTGTTTGAACTTGAACCTGCCTGACCTATGTGCACAATG  63913798

Query  3961      TTAACGTGTACAACATGAACTCCTGACATTGTCAGCAGGAATATGATGTTGCTTTGCTCA  4020
                 ||||||||||||||||||||||||||||||||||||||||||||||||||||||||||||
Sbjct  63913799  TTAACGTGTACAACATGAACTCCTGACATTGTCAGCAGGAATATGATGTTGCTTTGCTCA  63913858

Query  4021      TTTGAGTCTCAATTAGTTCACTGTGACATTACTATCCAATGTCTGTTAGCAGATGGTACT  4080
                 ||||||||||||||||||||||||||||||||||||||||||||||||||||||||||||
Sbjct  63913859  TTTGAGTCTCAATTAGTTCACTGTGACATTACTATCCAATGTCTGTTAGCAGATGGTACT  63913918

Query  4081      CAAATTATACTCAAATGAAGTTTTCTATAATCATATTAAGACACAGGGGTGGGGAAATGG  4140
                 ||||||||||||||||||||||||||||||||||||||||||||||||||||||||||||
Sbjct  63913919  CAAATTATACTCAAATGAAGTTTTCTATAATCATATTAAGACACAGGGGTGGGGAAATGG  63913978

Query  4141      GTCATGCAAAAATATTTACTAATACTACCTGTTCCATGTTCTTCCAGTTGTCTTCAAATC  4200
                 ||||||||||||||||||||||||||||||||||||||||||||||||||||||||||||
Sbjct  63913979  GTCATGCAAAAATATTTACTAATACTACCTGTTCCATGTTCTTCCAGTTGTCTTCAAATC  63914038

Query  4201      TCTGTAAGATTATATTGCTTCAAAATTCTTTGTGGCTTCAACATAGTGGATAACCAAAAA  4260
                 ||||||||||||||||||||||||||||||||||||||||||||||||||||||||||||
Sbjct  63914039  TCTGTAAGATTATATTGCTTCAAAATTCTTTGTGGCTTCAACATAGTGGATAACCAAAAA  63914098

Query  4261      GAGGCAAAATATGCCCACAGAACCTGATGGAAGAGTACATGTGATTCTCGCCATGCCATT  4320
                 ||||||||||||||||||||||||||||||||||||||||||||||||||||||||||||
Sbjct  63914099  GAGGCAAAATATGCCCACAGAACCTGATGGAAGAGTACATGTGATTCTCGCCATGCCATT  63914158

Query  4321      TCCCACAGACTAATCTAACCACGAATAGCTGTCACAGAAAGGATTGTTAAACGGTAACAC  4380
                 ||||||||||||||||||||||||||||||||||||||||||||||||||||||||||||
Sbjct  63914159  TCCCACAGACTAATCTAACCACGAATAGCTGTCACAGAAAGGATTGTTAAACGGTAACAC  63914218

Query  4381      TAAACACAAAAGGTCAATGTAGAAAAATCCATACTGGACACTCTAAAATAATGCGGAATC  4440
                 ||||||||||||||||||||||||||||||||||||||||||||||||||||||||||||
Sbjct  63914219  TAAACACAAAAGGTCAATGTAGAAAAATCCATACTGGACACTCTAAAATAATGCGGAATC  63914278

Query  4441      ATACCAATCTACATTAACTAGTTATTTTTGTCTTCACATAATTTCTTCTATTATACATTA  4500
                 ||||||||||||||||||||||||||||||||||||||||||||||||||||||||||||
Sbjct  63914279  ATACCAATCTACATTAACTAGTTATTTTTGTCTTCACATAATTTCTTCTATTATACATTA  63914338

Query  4501      CAGTATTATGGTCAGATTTTTCCAAGTCTGGGCCTAATGGATAGAAATTGAAGTTCTAAA  4560
                 ||||||||||||||||||||||||||||||||||||||||||||||||||||||||||||
Sbjct  63914339  CAGTATTATGGTCAGATTTTTCCAAGTCTGGGCCTAATGGATAGAAATTGAAGTTCTAAA  63914398

Query  4561      TTCTTCTATAAAGTTCAGATATTCGCTGATGCACTTTGATTCCCTCTCTGCCATTCCACG  4620
                 ||||||||||||||||||||||||||||||||||||||||||||||||||||||||||||
Sbjct  63914399  TTCTTCTATAAAGTTCAGATATTCGCTGATGCACTTTGATTCCCTCTCTGCCATTCCACG  63914458

Query  4621      AGTATCACAAGGCAGGTTCGTCAGCGTAAAGTCCTAAGAGTTATCAGAACCATTTTGTCT  4680
                 ||||||||||||||||||||||||||||||||||||||||||||||||||||||||||||
Sbjct  63914459  AGTATCACAAGGCAGGTTCGTCAGCGTAAAGTCCTAAGAGTTATCAGAACCATTTTGTCT  63914518

Query  4681      GACTGCATGGCAGTAACGTCTCAGCAAGTGGGCGATAAAGGATAGAACGGCAGCAACAGC  4740
                 ||||||||||||||||||||||||||||||||||||||||||||||||||||||||||||
Sbjct  63914519  GACTGCATGGCAGTAACGTCTCAGCAAGTGGGCGATAAAGGATAGAACGGCAGCAACAGC  63914578

Query  4741      AACATTGTAAGCTAAAAAGAAACAAGTTGCTAATAATATATTTAACCTTTATGAACAGAA  4800
                 ||||||||||||||||||||||||||||||||||||||||||||||||||||||||||||
Sbjct  63914579  AACATTGTAAGCTAAAAAGAAACAAGTTGCTAATAATATATTTAACCTTTATGAACAGAA  63914638

Query  4801      CTTATTGTTTGAGCAATTTCAGTCGGTTTTAACCTCAAAAACTCCTATCTATAAGACATA  4860
                 ||||||||||||||||||||||||||||||||||||||||||||||||||||||||||||
Sbjct  63914639  CTTATTGTTTGAGCAATTTCAGTCGGTTTTAACCTCAAAAACTCCTATCTATAAGACATA  63914698

Query  4861      GAATCGGTTCAGATTAGTTTAGTCTATAATTAGTTGACTGAGTTCCCTTAGTTCAGAATT  4920
                 ||||||||||||||||||||||||||||||||||||||||||||||||||||||||||||
Sbjct  63914699  GAATCGGTTCAGATTAGTTTAGTCTATAATTAGTTGACTGAGTTCCCTTAGTTCAGAATT  63914758

Query  4921      ACTAAAATGTAAATTCACTTTATGTTGTTTCTTCGAGTAATTGAGTGGAGAAGACTTCGA  4980
                 ||||||||||||||||||||||||||||||||||||||||||||||||||||||||||||
Sbjct  63914759  ACTAAAATGTAAATTCACTTTATGTTGTTTCTTCGAGTAATTGAGTGGAGAAGACTTCGA  63914818

Query  4981      TCCAATGGATTTCTTGGTTTCCTGTA-GGAATGTTAACAATCTGTGTTATACACTATT-A  5038
                 |||||||||||||||||||||||||| ||||||||||||||||||||||||||||||| |
Sbjct  63914819  TCCAATGGATTTCTTGGTTTCCTGTATGGAATGTTAACAATCTGTGTTATACACTATTGA  63914878

Query  5039      GATGCAGACG  5048
                 ||||||||||
Sbjct  63914879  GATGCAGACG  63914888
```

[Back to the top]

### Example 2. Micro-synteny of two genes between *Medicago truncatula* and soybean

[Back to the top]

**A. Two *Medicago truncatula* proteins used as query**

```
>IMGA|AC146590_10.2 AC146590.25 50435-49430 H EGN_Mt050401 20050623  SWIM zinc finger, putative
MKLMSPLQEQAHSDLTRFSFQKFQEEFVRSSQYSIDHENGNVFVVRFYKDVNSRKHVVFW
DGKVATCSCKLFEFWGILCRHILSIFLHKDCHEIPSNYLPSRWLLQVSYDDNDVESQVNV
VGEEQLLDCNNEPQPQHVVYCPPKSKPKGRPKRRRLKGGKELSHNMNTCGLCRGL
>IMGA|AC146590_11.2 AC146590.25 52557-50736 H EGN_Mt050401 20050623  hypothetical protein
MYVHVLSISRIKSPKETFPPLKNWKKKRKNCLLPNMGQEHIFWSSASCFDWYQKYGDVVV
FDTTYKVNSYEMPFGIFVDMNNYGKTILFGCALLRNEMVSAFRWLMKKPPTTILTNQDPW
MKEAISKEFPSKKHSFWIWHITFKFTSWFNALLRDKYAKWCSEFYELYKLETCEEFEHQW
PCC
```

[Back to the top]

**B. Parameters being used**

```
        Database: Glycine_max_WGS
        Initial E-value: 1e-60
        Round E-value: 1e-60
        Round Limit: 4
        Max Queries: 50
        Initial BLAST
                Percent Identity: none
                Filters: Low Complexity, Mask for lookup table only
        Recursive BLAST
                Word Size: 32
                Percent Identity: none
                Filters: Low Complexity, Rodents Repeats, Mask for lookup table only
        GenomeThreader Species: arabidopsis
```

[Back to the top]

**C. The produced contig that covers the soybean homologs of the above two Medicago proteins**

```
>Contig
AGCTTGCATGCCTGCAGGTCGACTCTAGAGGATCCCCTTTAATTTAATGAGGTACATATT
ATCCAATCATATAATCGTTTGATTCACATATTTTATTGTACATCATTTCTTTTATTTTGA
ACAATATCAAATAATATATAATATTAACACGTTATTTTAAAATCATACTTTATTAACTAC
ATGTACATGTAGTTCTATATTAATTATTCAAACTTTTCAATCAACTAGGAATATCAGCAC
GGGCATCTCACTAGGGTGTTTTTAAATGTGAATACTTGTTATTTTCAATAGTCATTATTT
TGAATGTATGACCGACACATATACTTAAACAGTACAATATATTTGATTAAGATTTTTGTG
CATATTTAAATATTAAAAACTTATAAATATAAAATAGTATTTTATTTTTAGATCATTATT
GTTTTAAAGTTATAATATTGTATTTTAAAATATTTTTATTATGCATTATTTAGTTATTAT
TATTATTATTATTATTATTATTATTTTAATATTATTGTTGTTATGTATTAGAAGTTTTCA
TTTATCTATTTATGTTAAAAATATCAAATAATATGAACAGGCTCAAGACTGAAGACAAAT
GGTTTAACATAATTGATAAATAATTATATTTACTAAGCATATTGACAGGTATTTGATTTC
TTGATATGGGTGCTGTTAACATGTGCCCTTAGGGCACATGTTAATGTGTAATAAATGATG
GTTGGTCCCCACTTATTATACTTTGGAAAAAACAGCATCATAAATGTGTCTCATAAAAAA
TTTAAAGACAAAATTACCCTCAACTATATGACACACATAGTGTGCTTTTGAACTTCCTAC
TTTAAATACACGCGCCTCAAAATTGCTAAATTGAAGAGTTTCTTTTTTTCTGACTTTTTT
GTCTCTTTACTGAGTATGAAGCACCTGCGTAGCATCTTTTGCAAAATAAAAGTATTTTTT
CATCCCTTCATTAACATTGCTGGAATTTTTTAATTTAAATAAATAATAAAAACTAATTCA
AAATATATTAGCAATTCAAATGAAATAATACCATTCCAATACAAAAAATATCCAAAAAGA
GGTTCAAATGTTTAATTAAAATAAAAAACTCATAAAAAATAGATCCAGGGCCTTCATGCC
TATGCCAACATCAGCTTTCTTTTCTAAACTCTGAAGTATCATTCCACATGCTACATTGCC
AACATATTCCTGTTAGAAACAAAGGGTAGAAACAAACAAAATATTATTAATTATCGGATA
TACATAATCCTACAAAAGTATATTAAACTAGAAATCTAAAGAATTTAATAAATGAAATGA
AAAAATATGTACCTAAACTTTTGGAAGAAGAATTGGATTTAAATTTGCATCTTTACAAAT
TTTTTTCTTCTTGTTTGTGTGACCAGAAAATTGAGTATTTTCTTTTAGAGGACATGTAAC
AATATTATGTCCTACATCTTTGCACAATCCACAAGTATTCATGTTATGTGATAGCTCTTT
TCCACCTTTAAGACGTCTTCGTTTGGGACGACCTTTTGTTTTGGAAGGTGGAGGACAGTG
AACAACATCGACTTGTTCCTCAAAGACAACATTGACTTGTTGGGGATCTACTTCATCATC
GTCATGTGATGTTTGAAGCCGCCATCGTGACGGCAAGTAGTTAGAAGGGATTTCATGACA
ATCTTTATGAAGAAAAATACTGAGAATGTGACGACATAATATACCCCAAAACTCAAAATA
CTTGCAACTACATGTAGCTATTTTACCATCCCAAAAGACCATGTGTTTTCTACTATTAGC
ATCTTTATAATACCGCAACACAAATTCATTACCATTTTCATGATGAATTGAATATTGGGT
GGACCTTTCAAACTCCTCTTGAAACTTCTGAAAAGCAAATCTTGTAAGAACACCATGAGC
TTGCTCTTGCAAGGGTGACATAAGCTTCAAGTTAATTCTTTTGCACTTTTCTAACATTAT
GTCATGGTCTTCTTTTTGCTTAATATCATCAATCGCTACATCAACCTGAAATTAATTTAA
ATTAATCAACGTATAAGATTAGAAGTGAAAAGTACTAATATATTGGCAATTACACCATAC
CTGCTTTGTAAAGTCACTTAAACTTGTGTGAGAATTTATAAATCTTTTAATAAATGCATT
AATACTCTCTGATCTTCCGGTAGTTGTCATCCCACCAAAGAAATGGTCACGAAGATAAGC
GAGTGCCCAGTAATTCCTGATTTCATACAATCCTTTCACATGTTTATTTGACTGCAAGTT
ATACTTAGCAACAACTTTTGGCCATTGATGCTCAAATTCTTCACATGTCTCCAATTTATA
CAACTCGTAAAAATCAGAACACCATTTTGAATATTTGTCCCGAAGTATAGCATTAAACCA
ACTACTAAACTTAAAAGTGATGTGCCATATGCAAAAACTATGTTTTGTTGATGGCAAGTC
TTTTGAAATTGCTTCTTTCATCCATGGATCTTGATCCGTTAGTATAGTCTTTGGTGGCTT
CTTCATCAAAGATATAAAAGTCTAAAGAAAATTTAGAAGCGTTAAAAAAAGTATTAATTA
GAAACTAAGATACCATCATTAAAGAAACAATAATAATAAGCTTAGAAGTTACCTTCATTA
ACCAACGAAATGCAGATATTGTTTCATTTCGTAAGAGTGCACAACCAAAAAGTACAGTCT
TCCCATGACTATTCATACCCACAAAAATCCCAAATGGCATTTCATAAGAATTGACCTTGT
ATGTAGTATCAAACACAACAACATCACCATATTTTTGGTACCAATCAGAGCAAGAAGCAG
GAGACCAAAAAATATGCTCTAACCTTCTCTCTTCATCAAGTGTATATGCATACTGAAATT
TAGAGCAACTCTTTTTTGCATCCTCACAGTACTTGAGAAGATCTTTGGCATCATTTCTTT
CAACTTTTTTCTTGGTTTTCACAAAAAGATTACGAATGTCCCTTTCAATAAATGGAAGAT
AACCATGCTTCACATTTTTCTCTAATTCTATGACACGCATCAATTGTCTAACTGAAAGCC
CCCCTTCTTTTAACAAGAAAATGCGTTCAATATCATCTTCAGAGATAGTTCGGTTAGCCG
GTAAAAAACGCACTTCTGATTGGGTTAGCAAAACATGGTTATGTTCAACAACAAACTTTG
TAACTCGCCACTCACTTGGAAATATATCATGAGACTTCTGCAATGAAATTCTTAAATAAG
CTTTACATTCACACCTTGTTGATTCTCTGTTTCTCTGTTCTTTTAATGGTTCTATGATTT
TCAACGAGCTTCTACCTTCACGATGGCAAAAAAAATCACGCCTCCTCATAATTCCATTTC
GTTTGATGAATCTACCTTTTCGAACAGAGAACCCATGCTGATATGCATACCTCTTATAAA
ATGCAAATGCTTCTTCCTCGCTAAGAAAAATTTGACCAACAAAAGGAACAATATTTGTCT
CATTATAAGTTTCATCTCTATCAACTTCTTCTGCAAATTCATTTATCTCAATATTCTGAC
TAGAAGACACAATATTTTCTTTCTCATAAGTTTCAATTTCCTCAACTTCTAATACATCAT
TTGCCACAATATCATGATCAAAATGTTCACTAACTGTTCCTGAATAACCATTCTCTAAAG
AAATATCTTCAATTGGATATTCATTTAAATCAACATTTATTTTATCGGTGCTCATATCTC
TAGTACTAGTACTACCTTCCCCCATTGTGTTATTTATGGTGCTCATATCTCTAGTACTAG
TATTACCTTCACCCATTGTGTTATTTATCATCTACAAATAAAAATAATAAATAGAAAAAT
ATATTACAAAACTCTATATATATATATATATATATATATATATATATATTTTGCAAGTGT
CCAAAAAACACATGTGATTGGCAAGAGCAATGGAGAAAAAAAAAGCTTGATAAAAGTACC
TGATATTGTGCCTTTGAGCTACGAAGAAAAGACTGTGCATGTGGGCTTTGAAGAAAAGAC
AAATCCATGGTTAATAATTCAATCTATCAATGTGTTTTTCATAAGAGCATATGGTGGTAG
AGAGGAATTGAAAAGAAATGTGCAAAGTGGTGGGAGTAGTACAGACTCTCGAACAAATTA
GCGGGAAACTAACAAAATCTAGGAAGAAAATA
```

[Back to the top]

**D. Pairwise alignment between the original Medicago query sequences and the above contig**

```
Query= IMGA|AC146590_10.2 H EGN_Mt050401 20050623  SWIM zinc finger, putative
         (175 letters)

 Score =  283 bits (725), Expect = 2e-78
 Identities = 135/174 (77%), Positives = 149/174 (85%), Gaps = 1/174 (0%)
 Frame = -3

Query: 1    MKLMSPLQEQAHSDLTRFSFQKFQEEFVRSSQYSIDHENGNVFVVRFYKDVNSRKHVVFW 60
            +KLMSPLQEQAH  LTRF+FQKFQEEF RS+QYSI HENGN FV+R+YKD NSRKH+VFW
Sbjct: 1950 LKLMSPLQEQAHGVLTRFAFQKFQEEFERSTQYSIHHENGNEFVLRYYKDANSRKHMVFW 1771

Query: 61   DGKVATCSCKLFEFWGILCRHILSIFLHKDCHEIPSNYLPSRWLLQVSYDDNDVE-SQVN 119
            DGK+ATCSCK FEFWGILCRHILSIFLHKDCHEIPSNYLPSRW LQ S+DD++V+  QVN
Sbjct: 1770 DGKIATCSCKYFEFWGILCRHILSIFLHKDCHEIPSNYLPSRWRLQTSHDDDEVDPQQVN 1591

Query: 120  VVGEEQLLDCNNEPQPQHVVYCPPKSKPKGRPKRRRLKGGKELSHNMNTCGLCR 173
            VV EEQ+           VV+CPP SK KGRPKRRRLKGGKELSHNMNTCGLC+
Sbjct: 1590 VVFEEQV----------DVVHCPPPSKTKGRPKRRRLKGGKELSHNMNTCGLCK 1459


Query= IMGA|AC146590_11.2 H EGN_Mt050401 20050623  hypothetical
protein
         (183 letters)

 Score =  157 bits (396), Expect(2) = 4e-75
 Identities = 67/77 (87%), Positives = 73/77 (94%)
 Frame = -3

Query: 105  LMKKPPTTILTNQDPWMKEAISKEFPSKKHSFWIWHITFKFTSWFNALLRDKYAKWCSEF 164
            LMKKPP TILT+QDPWMKEAISK+ PS KHSF IWHITFKF+SWFNA+LRDKY+KWCS+F
Sbjct: 2529 LMKKPPKTILTDQDPWMKEAISKDLPSTKHSFCIWHITFKFSSWFNAILRDKYSKWCSDF 2350

Query: 165  YELYKLETCEEFEHQWP 181
            YELYKLETCEEFEHQWP
Sbjct: 2349 YELYKLETCEEFEHQWP 2299


 Score =  137 bits (346), Expect(2) = 4e-75
 Identities = 61/69 (88%), Positives = 65/69 (94%)
 Frame = -2

Query: 39   EHIFWSSASCFDWYQKYGDVVVFDTTYKVNSYEMPFGIFVDMNNYGKTILFGCALLRNEM 98
            EHIFWS ASC DWYQKYGDVVVFDTTYKVNSYEMPFGIFV MN++GKT+LFGCALLRNE 
Sbjct: 2839 EHIFWSPASCSDWYQKYGDVVVFDTTYKVNSYEMPFGIFVGMNSHGKTVLFGCALLRNET 2660

Query: 99   VSAFRWLMK 107
            +SAFRWLMK
Sbjct: 2659 ISAFRWLMK 2633
```

[Back to the top]

**E. Spliced alignment between the above contig and the two query protein sequences**

```
********************************************************************************
Protein Sequence: file=/PlantGDB/html/tmp/tracembler-1167778359/QRY.fsa, description=IMGA|AC146590_10.2 H EGN_Mt050401 20050623  SWIM zinc finger, putative

    1  MKLMSPLQEQ AHSDLTRFSF QKFQEEFVRS SQYSIDHENG NVFVVRFYKD VNSRKHVVFW
   61  DGKVATCSCK LFEFWGILCR HILSIFLHKD CHEIPSNYLP SRWLLQVSYD DNDVESQVNV
  121  VGEEQLLDCN NEPQPQHVVY CPPKSKPKGR PKRRRLKGGK ELSHNMNTCG LCRGL

Genomic Template: file=/PlantGDB/html/tmp/tracembler-1167778359/reads.cap.contigs, strand=-, from=2247, to=1162, description=Contig

Predicted gene structure:

 Exon  1 1950 1453 ( 498 n);  Protein      1    175 ( 175 aa); score: 0.637

MATCH	Contig-	IMGA+	0.637	498	0.949	P
PGS_Contig-_IMGA+	(1950  1453)

Alignment (genomic DNA sequence = upper lines):

TTGAAGCTTA TGTCACCCTT GCAAGAGCAA GCTCATGGTG TTCTTACAAG ATTTGCTTTT        1891
 L  K  L   M  S  P  L   Q  E  Q   A  H  G   V  L  T  R   F  A  F 
 +  |  |   |  |  |  |   |  |  |   |  |  .      |  |  |   |  +  | 
 M  K  L   M  S  P  L   Q  E  Q   A  H  S   D  L  T  R   F  S  F           20


CAGAAGTTTC AAGAGGAGTT TGAAAGGTCC ACCCAATATT CAATTCATCA TGAAAATGGT        1831
 Q  K  F   Q  E  E  F   E  R  S   T  Q  Y   S  I  H  H   E  N  G 
 |  |  |   |  |  |  |      |  |   +  |  |   |  |     |   |  |  | 
 Q  K  F   Q  E  E  F   V  R  S   S  Q  Y   S  I  D  H   E  N  G           40


AATGAATTTG TGTTGCGGTA TTATAAAGAT GCTAATAGTA GAAAACACAT GGTCTTTTGG        1771
 N  E  F   V  L  R  Y   Y  K  D   A  N  S   R  K  H  M   V  F  W 
 |     |   |  +  |  +   |  |  |   .  |  |   |  |  |  +   |  |  | 
 N  V  F   V  V  R  F   Y  K  D   V  N  S   R  K  H  V   V  F  W           60


GATGGTAAAA TAGCTACATG TAGTTGCAAG TATTTTGAGT TTTGGGGTAT ATTATGTCGT        1711
 D  G  K   I  A  T  C   S  C  K   Y  F  E   F  W  G  I   L  C  R 
 |  |  |   +  |  |  |   |  |  |      |  |   |  |  |  |   |  |  | 
 D  G  K   V  A  T  C   S  C  K   L  F  E   F  W  G  I   L  C  R           80


CACATTCTCA GTATTTTTCT TCATAAAGAT TGTCATGAAA TCCCTTCTAA CTACTTGCCG        1651
 H  I  L   S  I  F  L   H  K  D   C  H  E   I  P  S  N   Y  L  P 
 |  |  |   |  |  |  |   |  |  |   |  |  |   |  |  |  |   |  |  | 
 H  I  L   S  I  F  L   H  K  D   C  H  E   I  P  S  N   Y  L  P          100


TCACGATGGC GGCTTCAAAC ATCACATGAC GATGATGAAG TAGATCCCCA ACAAGTCAAT        1591
 S  R  W   R  L  Q  T   S  H  D   D  D  E   V  D     P   Q  Q  V 
 |  |  |      |  |  .   |  +  |   |  +  +   |  +         |       
 S  R  W   L  L  Q  V   S  Y  D   D  N  D   V  E     S   Q  V  N          120


GTTGTCTTTG AGGAACAAGT C---GAT--- ---------- ---------- ----GTTGTT        1561
 N  V  V   F  E  E  Q      V                                D  V 
    |         |  +                                             | 
 V  V  G   E  E  Q  L   L  D  C   N  N  E   P  Q  P  Q   H  V  V          140


CACTGTCCTC CACCTTCCAA AACAAAAGGT CGTCCCAAAC GAAGACGTCT TAAAGGTGGA        1501
 V  H  C   P  P  P  S   K  T  K   G  R  P   K  R  R  R   L  K  G 
           |        .                       +  |  |            | 
 Y  C  P   P  K  S  K   P  K  G   R  P  K   R  R  R  L   K  G  G          160


AAAGAGCTAT CACATAACAT GAATACTTGT GGATTGTGCA AAGATGTA                     1453
 G  K  E   L  S  H  N   M  N  T   C  G  L   C  K  D 
    +            +         .                        
 K  E  L   S  H  N  M   N  T  C   G  L  C   R  G  L                       176


********************************************************************************
Protein Sequence: file=/PlantGDB/html/tmp/tracembler-1167778359/QRY.fsa, description=IMGA|AC146590_11.2 H EGN_Mt050401 20050623  hypothetical protein

    1  MYVHVLSISR IKSPKETFPP LKNWKKKRKN CLLPNMGQEH IFWSSASCFD WYQKYGDVVV
   61  FDTTYKVNSY EMPFGIFVDM NNYGKTILFG CALLRNEMVS AFRWLMKKPP TTILTNQDPW
  121  MKEAISKEFP SKKHSFWIWH ITFKFTSWFN ALLRDKYAKW CSEFYELYKL ETCEEFEHQW
  181  PCC

Genomic Template: file=/PlantGDB/html/tmp/tracembler-1167778359/reads.cap.contigs, strand=-, from=3106, to=1999, description=Contig

Predicted gene structure:

 Exon  1 3105 3053 (  53 n);  Protein     23     39 (  17 aa); score: 0.211
  Intron  1 3052 2837 ( 216 n); Pd: 0.050   Pa: 0.050 
 Exon  2 2836 2633 ( 204 n);  Protein     40    107 (  68 aa); score: 0.886
  Intron  2 2632 2521 ( 112 n); Pd: 0.050   Pa: 0.050 
 Exon  3 2520 2291 ( 230 n);  Protein    108    183 (  76 aa); score: 0.832

MATCH	Contig-	IMGA+	0.790	487	0.887	P
PGS_Contig-_IMGA+	(3105  3053,2836  2633,2520  2291)

Alignment (genomic DNA sequence = upper lines):

TCTCTGAAGA TGA-TATTGA ACGCATTTTC TTGTTAAAAG AAGGGGGGCT TTCAGTTAGA        3047
 S  L  K   M     I  E   R  I  F   L  L  K   E  G  G  L   S       
 +     |            .   +         |  |      .     |      .       
 N  W  K   K     K  R   K  N  C   L  L  P   N  M  G  Q   E ......          40


CAATTGATGC GTGTCATAGA ATTAGAGAAA AATGTGAAGC ATGGTTATCT TCCATTTATT        2987
                                                                 
                                                                 
.......... .......... .......... .......... .......... ..........          40


GAAAGGGACA TTCGTAATCT TTTTGTGAAA ACCAAGAAAA AAGTTGAAAG AAATGATGCC        2927
                                                                 
                                                                 
.......... .......... .......... .......... .......... ..........          40


AAAGATCTTC TCAAGTACTG TGAGGATGCA AAAAAGAGTT GCTCTAAATT TCAGTATGCA        2867
                                                                 
                                                                 
.......... .......... .......... .......... .......... ..........          40


TATACACTTG ATGAAGAGAG AAGGTTAGAG CATATTTTTT GGTCTCCTGC TTCTTGCTCT        2807
                                  H  I  F   W  S  P  A   S  C  S 
                                  |  |  |   |  |     |   |  |    
.......... .......... ..........  H  I  F   W  S  S  A   S  C  F           50


GATTGGTACC AAAAATATGG TGATGTTGTT GTGTTTGATA CTACATACAA GGTCAATTCT        2747
 D  W  Y   Q  K  Y  G   D  V  V   V  F  D   T  T  Y  K   V  N  S 
 |  |  |   |  |  |  |   |  |  |   |  |  |   |  |  |  |   |  |  | 
 D  W  Y   Q  K  Y  G   D  V  V   V  F  D   T  T  Y  K   V  N  S           70


TATGAAATGC CATTTGGGAT TTTTGTGGGT ATGAATAGTC ATGGGAAGAC TGTACTTTTT        2687
 Y  E  M   P  F  G  I   F  V  G   M  N  S   H  G  K  T   V  L  F 
 |  |  |   |  |  |  |   |  |      |  |  +   +  |  |  |   +  |  | 
 Y  E  M   P  F  G  I   F  V  D   M  N  N   Y  G  K  T   I  L  F           90


GGTTGTGCAC TCTTACGAAA TGAAACAATA TCTGCATTTC GTTGGTTAAT GAAGGTAACT        2627
 G  C  A   L  L  R  N   E  T  I   S  A  F   R  W  L  M   K       
 |  |  |   |  |  |  |   |     +   |  |  |   |  |  |  |   |       
 G  C  A   L  L  R  N   E  M  V   S  A  F   R  W  L  M   K ......         108


TCTAAGCTTA TTATTATTGT TTCTTTAATG ATGGTATCTT AGTTTCTAAT TAATACTTTT        2567
                                                                 
                                                                 
.......... .......... .......... .......... .......... ..........         108


TTTAACGCTT CTAAATTTTC TTTAGACTTT TATATCTTTG ATGAAGAAGC CACCAAAGAC        2507
                                                   K   P  P  K  T
                                                   |   |  |     |
.......... .......... .......... .......... ...... K   P  P  T  T         113


TATACTAACG GATCAAGATC CATGGATGAA AGAAGCAATT TCAAAAGACT TGCCATCAAC        2447
  I  L  T   D  Q  D   P  W  M  K   E  A  I   S  K  D   L  P  S  T
  |  |  |   +  |  |   |  |  |  |   |  |  |   |  |  +   .  |  |   
  I  L  T   N  Q  D   P  W  M  K   E  A  I   S  K  E   F  P  S  K         133


AAAACATAGT TTTTGCATAT GGCACATCAC TTTTAAGTTT AGTAGTTGGT TTAATGCTAT        2387
  K  H  S   F  C  I   W  H  I  T   F  K  F   S  S  W   F  N  A  I
  |  |  |   |     |   |  |  |  |   |  |  |   +  |  |   |  |  |  +
  K  H  S   F  W  I   W  H  I  T   F  K  F   T  S  W   F  N  A  L         153


ACTTCGGGAC AAATATTCAA AATGGTGTTC TGATTTTTAC GAGTTGTATA AATTGGAGAC        2327
  L  R  D   K  Y  S   K  W  C  S   D  F  Y   E  L  Y   K  L  E  T
  |  |  |   |  |  +   |  |  |  |   +  |  |   |  |  |   |  |  |  |
  L  R  D   K  Y  A   K  W  C  S   E  F  Y   E  L  Y   K  L  E  T         173


ATGTGAAGAA TTTGAGCATC AATGGCCAA- AAGTTGT                                 2291
  C  E  E   F  E  H   Q  W  P      S  C 
  |  |  |   |  |  |   |  |  |         | 
  C  E  E   F  E  H   Q  W  P      C  C                                   185


--------------------------------------------------------------------------------
```

[Back to the top]
